# Artificial intelligence-based tongue image analysis for non-invasive screening of gastric cancer, precancerous lesions, and related gastrointestinal disorders: a systematic review

**DOI:** 10.3389/fmed.2026.1931256

**Published:** 2026-09-29

**Authors:** Rukai Wang, Chunlin Li, Hengyan Zhang, Pingping Shao, Zhen Gao, Chenyue Zhang

**Affiliations:** 1 First School of Clinical medicine, Shandong University of Traditional Chinese Medicine, Jinan, China; 2 Department of Neurology II, Shandong University of Traditional Chinese Medicine Affiliated Hospital, Jinan, China

**Keywords:** artificial intelligence, deep learning, gastric cancer, machine learning, non-invasive screening, precancerous gastric lesions, tongue image analysis, traditional Chinese medicine

## Abstract

**Background:**

Gastric cancer remains a major cause of cancer-related mortality, largely because many cases are diagnosed at an advanced stage. Upper gastrointestinal endoscopy with histopathological confirmation is the diagnostic gold standard, but its invasiveness, cost, and limited accessibility restrict population-level use. Artificial intelligence (AI)-based tongue-image analysis may provide an objective, low-cost, and non-invasive adjunct for pre-endoscopic risk stratification.

**Methods:**

This systematic review was conducted according to PRISMA 2020 principles and was informed by diagnostic test accuracy and prediction-model reporting frameworks. PubMed/MEDLINE, Scopus, Web of Science, and IEEE Xplore were searched from inception to 10 June 2026. Eligible studies were original investigations applying machine-learning or deep-learning methods to digital tongue images for gastric cancer, precancerous gastric lesions, or related gastrointestinal disorders and reporting at least one quantitative performance metric. Data were extracted on study design, sample size, target condition, reference standard, image-acquisition protocol, model architecture, validation strategy, diagnostic performance, calibration, interpretability, and risk of bias. A narrative synthesis was performed because of substantial heterogeneity.

**Results:**

The search identified 139 records, of which 78 remained after duplicate removal. After title, abstract, and full-text screening, 9 studies published between 2018 and 2025 were included. The evidence covered gastric cancer, precancerous gastric lesions or high-risk gastric screening, and related gastrointestinal disorders. Models included logistic regression and XGBoost classifiers based on handcrafted features, convolutional neural networks, transformer-based architectures, segmentation-assisted pipelines, and multimodal models combining tongue images with clinical, questionnaire, biomarker, or microbiome data. Internally validated AUCs ranged from 0.61 to 0.99 across target conditions. External validation was reported in only three studies and generally showed performance degradation, particularly for precancerous gastric lesions. Calibration assessment was uncommon, and most studies did not report calibration curves, Brier scores, expected calibration error, or recalibration procedures.

**Conclusion:**

AI-based tongue-image analysis is a promising but early-stage approach for non-invasive gastric disease screening. Current evidence supports its potential role as an adjunctive pre-endoscopic triage tool rather than a replacement for gastroscopy. Prospective external validation, standardized image acquisition, transparent model reporting, calibration assessment, and evaluation in diverse populations are required before clinical deployment.

**Systematic Review Registration:**

https://www.crd.york.ac.uk/PROSPERO/view/CRD420261444857, PROSPERO CRD420261444857.

## 1 Introduction

Gastric cancer remains a major global health burden and continues to be associated with substantial cancer-related mortality. According to GLOBOCAN 2022 estimates, gastric cancer is the fifth most common cancer and the fifth leading cause of cancer death worldwide, with approximately 969,000 new cases and 660,000 deaths annually (1). A major contributor to this burden is late-stage diagnosis. Many patients are diagnosed only after the disease has progressed beyond a curable stage, when treatment options become more limited and survival outcomes decline substantially (2). Although upper gastrointestinal endoscopy with histopathological confirmation remains the diagnostic gold standard, its use as a population-level screening tool is constrained by cost, invasiveness, patient discomfort, the need for trained endoscopists, and limited accessibility in low-resource or rural settings. These limitations have created a need for inexpensive, scalable, and non-invasive tools that can identify individuals who may benefit most from confirmatory endoscopic assessment.

Tongue diagnosis has long been used in traditional Chinese medicine as part of clinical assessment, particularly for disorders involving the spleen and stomach. Classical tongue inspection evaluates tongue body color, coating color and thickness, moisture, shape, fissures, tooth marks, and other visual features that are interpreted as reflections of internal physiological states (3). However, traditional tongue diagnosis is inherently subjective and can be influenced by practitioner experience, lighting conditions, image quality, and patient cooperation. Previous studies have reported only modest inter-rater consistency among practitioners, underscoring the need for objective and reproducible methods (4). Similar diagnostic traditions also exist outside Chinese medicine, including in Persian medicine, suggesting that visual inspection of the tongue has broader cross-cultural relevance as a clinical observation tool (5).

Recent advances in digital imaging, computer vision, machine learning, and deep learning have created an opportunity to transform tongue diagnosis from a qualitative clinical impression into a quantitative image-analysis task. Automated tongue-image systems can segment the tongue, extract color, texture, geometric, and coating-related features, and classify images according to disease status. Several reviews have examined AI-assisted tongue diagnosis in general (6–10), and others have discussed the broader intersection of traditional medicine and artificial intelligence (11, 12). However, the evidence specific to gastric cancer, precancerous gastric lesions, and related gastrointestinal disorders remains fragmented. In particular, there has been limited synthesis of how model performance varies according to study design, validation strategy, disease target, calibration reporting, and clinical applicability.

The biological rationale for tongue-image analysis in gastric disease is supported by both traditional diagnostic theory and emerging evidence for an oral–gastric axis. In traditional Chinese medicine, the central and posterior regions of the tongue are considered to correspond to the spleen and stomach, and digestive disorders are thought to manifest through changes in tongue coating, color, moisture, and morphology (13). Modern microbiome and metabolomic studies provide complementary evidence. Differences in tongue-coating microbiota and metaproteomic profiles have been reported between patients with gastric cancer, precancerous gastric lesions, chronic gastritis, and healthy controls (14–21). Associations between tongue coating and gastric fluid microbiomes further support the possibility that the tongue may reflect disease-associated changes within the upper gastrointestinal tract (22). Nevertheless, tongue appearance is also affected by diet, smoking, alcohol consumption, oral hygiene, medications, systemic disease, and environmental conditions; therefore, a plausible oral–gastric association does not establish diagnostic specificity. Across the nine included studies, smoking-related factors were addressed in 4/9 studies and dietary factors in 2/9, whereas oral hygiene was not reported as an adjustment factor in any study. Six studies addressed at least one major lifestyle-related factor through matching, exclusion, regression modelling, or incorporation of clinical variables into multimodal models. Importantly, however, adjustment through multimodal fusion should not be equated with demonstrating that image-derived features are independent of these confounders. Only limited evidence directly evaluated whether the learned visual representation remained robust after accounting for lifestyle and systemic factors. Thus, confounding remains a substantial unresolved limitation in the primary literature.

This systematic review therefore aimed to synthesize the available evidence on artificial intelligence-based tongue-image analysis for non-invasive screening of gastric cancer, precancerous gastric lesions, and related gastrointestinal disorders. Specifically, we sought to characterize the included studies, summarize image-acquisition and preprocessing approaches, compare machine-learning and deep-learning model architectures, evaluate diagnostic performance and validation strategies, assess risk of bias and calibration reporting, and identify the key methodological gaps that must be addressed before tongue-image AI can be considered for clinical implementation.

## 2 Methods

### 2.1 Reporting framework and protocol

This systematic review was conducted and reported in accordance with the PRISMA 2020 statement; the completed PRISMA 2020 checklist is provided in Supplementary Table S1. Because the included studies evaluated artificial intelligence, machine-learning, or deep-learning models for disease screening or classification, reporting and methodological appraisal were additionally informed by TRIPOD + AI and PROBAST-AI/PROBAST + AI principles (23, 24). The review methods, including the research question, eligibility criteria, information sources, data-extraction domains, risk-of-bias approach, and synthesis strategy, were defined before final study selection and data extraction.

The core review question, eligibility criteria, information sources, and primary outcome domains remained unchanged after PROSPERO registration (CRD420261444857). However, two methodological extensions were introduced during manuscript preparation and are explicitly reported here. First, the risk-of-bias assessment was operationalized in greater detail for AI-based diagnostic studies, including an explicit analysis domain and separate applicability judgments. Second, a structured evidence-certainty framework informed by GRADE principles was added *post hoc* to provide a transparent assessment of the strength of the heterogeneous evidence. The PROSPERO record had stated that formal certainty assessment would not be performed; therefore, the certainty framework is considered a *post hoc* methodological extension rather than a prespecified protocol component.

### 2.2 Review question and eligibility criteria

The review question was structured using a diagnostic-review framework. The population of interest comprised individuals evaluated for gastric cancer, precancerous gastric lesions, or related gastrointestinal disorders, including healthy or non-disease controls. The index test was any artificial intelligence, machine-learning, or deep-learning model applied to digital tongue images, either alone or in combination with clinical variables, questionnaire data, biomarkers, or microbiome information. Eligible reference standards included histopathology, gastroscopy, clinically accepted diagnostic criteria, or clearly defined study-specific diagnostic labels. The target conditions included gastric cancer, precancerous gastric lesions such as atrophic gastritis, intestinal metaplasia or dysplasia, and other related gastrointestinal disorders. Outcomes of interest included diagnostic or predictive performance metrics such as area under the receiver operating characteristic curve, sensitivity, specificity, accuracy, F1-score, calibration, external validation, and interpretability.

Original research studies were eligible if they applied machine-learning or deep-learning methods to tongue images for the diagnosis, screening, or risk classification of gastric cancer, precancerous gastric lesions, or related gastrointestinal disorders, and reported at least one quantitative performance metric. Review articles, editorials, case reports, conference abstracts without sufficient methodological detail, studies using only non-image tongue data, studies focused exclusively on tongue segmentation without disease classification, and studies unrelated to gastric or gastrointestinal disease targets were excluded.

### 2.3 Information sources and search strategy

A systematic literature search was performed in PubMed/MEDLINE, Scopus, Web of Science, and IEEE Xplore. The search covered studies published from inception to June 2026, and the final search was conducted on 10 June 2026. Search terms combined three concept groups: tongue imaging, artificial intelligence, and gastric or gastrointestinal disease. The tongue-imaging domain included terms such as “tongue image,” “tongue diagnosis,” and “tongue coating.” The artificial-intelligence domain included terms such as “artificial intelligence,” “machine learning,” “deep learning,” “convolutional neural network,” and “computer-aided diagnosis.” The disease domain included terms such as “gastric cancer,” “stomach cancer,” “precancerous gastric lesions,” and “gastrointestinal disease.” Boolean operators and database-specific syntax were used to adapt the strategy for each database. The full database-specific search strategies, including the complete search strings used for PubMed/MEDLINE, Scopus, Web of Science, and IEEE Xplore, are provided in Supplementary Tables S2, S3. Reference lists of included studies and relevant reviews were also manually screened to identify additional eligible articles.

### 2.4 Study selection

All retrieved records were imported into a reference-management system and duplicates were removed before screening. Titles and abstracts were screened independently by two reviewers (RW and CL). Full texts of potentially eligible studies were then assessed independently against the predefined eligibility criteria. Disagreements were resolved through discussion, and unresolved disagreements were adjudicated by a third reviewer (CZ). Cohen's kappa (*κ*) was calculated *post hoc* to quantify inter-reviewer agreement. Agreement was substantial for title/abstract screening (*κ* = 0.77) and full-text eligibility assessment (*κ* = 0.63). Reasons for exclusion at the full-text stage were recorded and summarized in the PRISMA flow diagram. (Figure 1).

**Figure 1:**
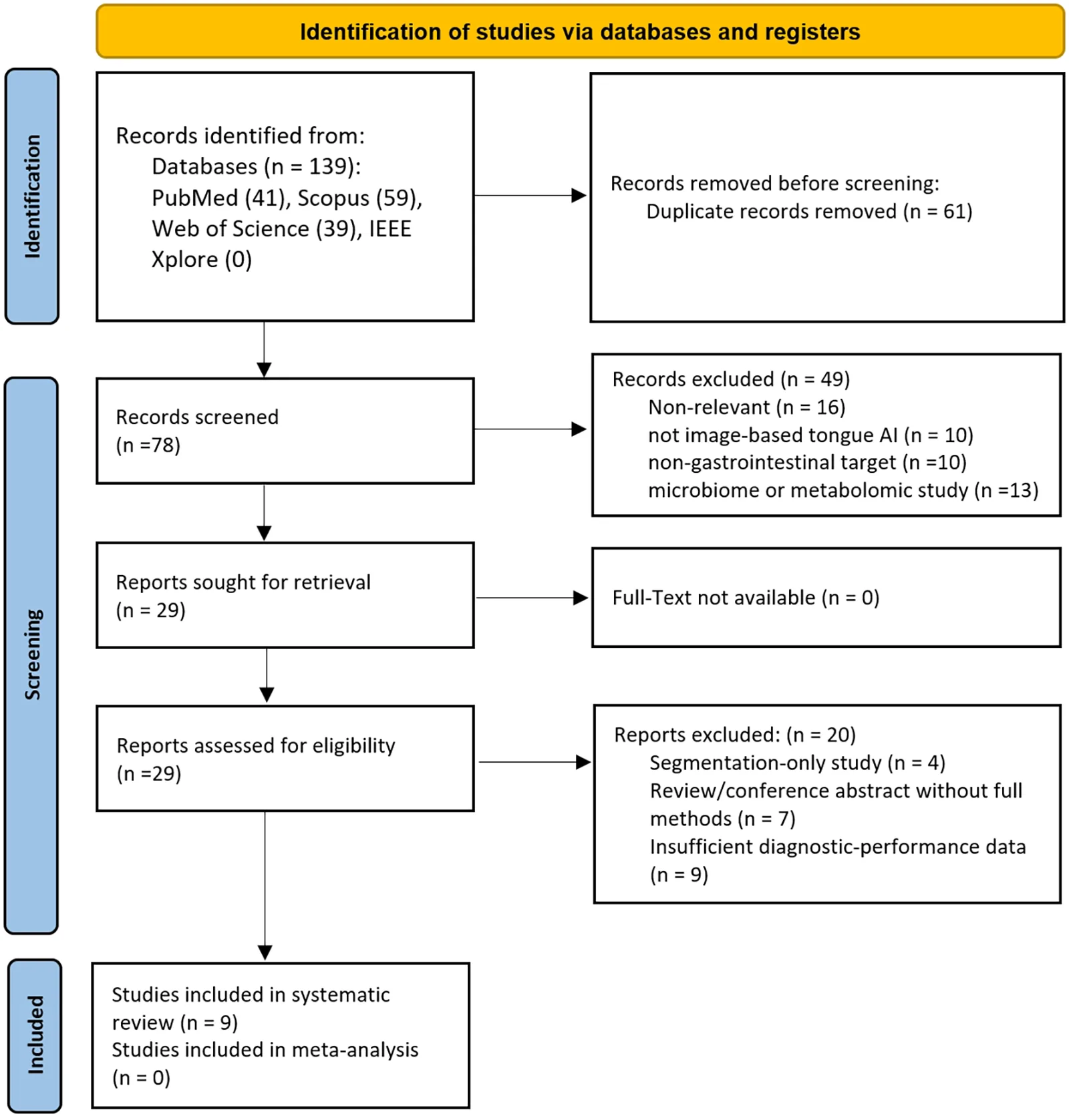
PRISMA flow diagram.

The database search identified 139 records: 41 from PubMed/MEDLINE, 59 from Scopus, 39 from Web of Science, and 0 from IEEE Xplore. After removal of 61 duplicate records, 78 records underwent title and abstract screening. Forty-nine records were excluded at this stage because they were non-relevant, did not apply image-based tongue AI, did not involve a gastrointestinal target, or used microbiome/metabolomic data without image-based AI. Twenty-nine reports were sought for retrieval, and all were available for full-text assessment. Of these, 20 reports were excluded because they were segmentation-only studies, review articles or conference abstracts without full methods, or did not report sufficient diagnostic-performance data. Nine studies met the eligibility criteria and were included in the final systematic review. No meta-analysis was performed.

### 2.5 Data extraction

Data were extracted using a predefined standardized form. Extracted items included first author, publication year, country, study design, number of centers, sample size, participant groups, target condition, reference standard, image-acquisition device and protocol, preprocessing and segmentation methods, feature-extraction approach, model architecture, training and validation strategy, use of external validation, reported performance metrics, calibration assessment, interpretability methods, and key findings. Data extraction was performed independently by two reviewers (RW and CL) and cross-checked for accuracy. Discrepancies were resolved by consensus. kappa (*κ*) for inter-reviewer agreement during data extraction was 0.73.

For diagnostic and predictive performance measures, 95% confidence intervals were extracted when reported in the primary studies. When confidence intervals were not reported and the published information was insufficient for reliable reconstruction, the estimate was retained as reported and the confidence interval was recorded as not reported. Confidence intervals were not calculated from incomplete or incompatible aggregate data.

### 2.6 Risk of bias and applicability assessment

Risk of bias and applicability were assessed using domains adapted from PROBAST-AI/PROBAST + AI and diagnostic test accuracy review principles (23, 25). The assessment considered participant selection, index test or predictor measurement, reference standard or outcome definition, flow and timing, and analysis. Participant selection was judged at lower risk when studies used prospective or consecutive recruitment and at higher risk when they used case-control sampling with healthy controls only. The index-test domain considered whether tongue-image acquisition, preprocessing, segmentation, and model application were sufficiently standardized and reproducible. The reference-standard domain considered whether disease status was verified using gastroscopy, histopathology, or other accepted clinical criteria. The analysis domain considered whether the study used an appropriate participant-level data-splitting strategy; whether data augmentation or resampling could introduce leakage between development and test datasets; whether the sample size and number of events were appropriate for the modelling approach; whether model development, tuning, and final performance assessment were appropriately separated; how class imbalance was handled; whether decision thresholds were appropriately determined; whether calibration was assessed; and whether independent external validation was performed. For each domain, judgments were classified as low, high, or unclear risk of bias. An unclear judgment was assigned when the published report did not provide sufficient information to determine whether the relevant methodological requirement had been satisfied. Applicability was assessed separately from risk of bias. Applicability judgments considered whether the study population, target condition, index test and image-acquisition approach, and reference standard were appropriate for the intended use of AI-based tongue-image analysis as a non-invasive pre-endoscopic screening or triage tool.

### 2.7 Data synthesis

A formal meta-analysis was not performed because of substantial clinical and methodological heterogeneity across the included studies. The studies differed in target condition and disease spectrum, participant selection, reference-standard definition, tongue-image acquisition and preprocessing, model architecture, input modalities, validation strategy, decision thresholds, and reported performance metrics. In particular, gastric cancer, precancerous gastric lesions, and other gastrointestinal disorders represented clinically distinct diagnostic targets and were not evaluated using comparable reference standards or populations. Several studies also evaluated multimodal models incorporating clinical variables, biomarkers, or microbiome data, making direct comparison with image-only models inappropriate. In addition, confidence intervals were inconsistently reported, and external validation was available for only a minority of studies.

Under these circumstances, statistical pooling of AUC, sensitivity, specificity, or accuracy was considered inappropriate because a pooled estimate could obscure clinically important differences between disease targets, study populations, reference standards, and model-development strategies and could imply a degree of comparability that was not supported by the underlying evidence. A structured narrative synthesis was therefore performed instead.

Studies were organized into three clinical evidence groups: (1) gastric cancer; (2) precancerous gastric lesions and high-risk gastric screening; and (3) contextual evidence from other gastrointestinal disorders. The third group included studies addressing GERD, MAFLD, and esophageal cancer or premalignant esophageal lesions. These studies were retained because related gastrointestinal disorders were included in the prespecified eligibility criteria, but their findings were not used to estimate or characterize the diagnostic performance of tongue-image AI specifically for gastric cancer.

Within each group, findings were summarized according to study design, sample size, target condition, reference standard, image-acquisition protocol, model architecture, input modality, validation strategy, discrimination performance, calibration, interpretability, and risk of bias. Greater interpretive weight was given to studies with prospective or multicenter recruitment, histopathologically verified disease, independent external validation, and transparent reporting.

### 2.8 Strength of evidence assessment

Because the included studies were heterogeneous in target condition, study design, reference standard, model architecture, validation strategy, and reported performance measures, a structured, modified GRADE-informed framework was used to assess the certainty of the available evidence. This assessment was added after protocol registration and is therefore considered a *post-hoc* methodological extension rather than a prespecified protocol component.

Five domains were considered: risk of bias, indirectness, imprecision, inconsistency, and external validity. Risk of bias was informed by the study-level assessment of participant selection, index test, reference standard, flow and timing, and analysis. Indirectness considered differences between the study population, target condition, index test, reference standard, and the intended use of tongue-image AI for non-invasive gastric disease screening or triage. Imprecision considered sample size, event numbers, and the availability and width of 95% confidence intervals for key performance estimates. Inconsistency was considered when interpreting findings across studies addressing comparable clinical questions. External validity considered independent or external validation, multicenter evaluation, and diversity of populations and image-acquisition settings.

Study-level judgments for risk of bias, indirectness, imprecision, and external validity were summarized for each included study. Overall certainty was then judged for the three evidence groups: gastric cancer, precancerous gastric lesions/high-risk gastric screening, and contextual evidence from other gastrointestinal disorders. Evidence was categorized as high, moderate, low, or very low certainty, with downgrading applied when important limitations were identified across the relevant domains. Detailed judgments and their rationales are provided in Supplementary Table S4.

Because no quantitative meta-analysis was performed and the evidence base consisted of a small number of heterogeneous studies, these certainty ratings characterize the confidence in the overall evidence rather than representing pooled estimates of diagnostic accuracy.

## 3 Results

### 3.1 Study selection

The study-selection process is summarized in Figure 1. The database search identified 139 records, including 41 from PubMed/MEDLINE, 59 from Scopus, 39 from Web of Science, and none from IEEE Xplore. After removal of 61 duplicate records, 78 records were screened by title and abstract. Forty-nine records were excluded at this stage: 16 were non-relevant, 10 did not apply image-based tongue AI, 10 did not involve gastrointestinal disease targets, and 13 were microbiome or metabolomic studies without image-based classification. Twenty-nine reports were sought for retrieval, and no full text was unavailable. All 29 reports were assessed for eligibility. Twenty reports were excluded after full-text review: 4 were segmentation-only studies, 7 were reviews or conference abstracts without full methods, and 9 had insufficient diagnostic-performance data. Nine original studies were included in the final systematic review, and none were included in a meta-analysis.

### 3.2 Characteristics of included studies

The characteristics of the included studies are summarized in Table 1. The 9 studies were published between 2018 and 2025 and were conducted predominantly in East Asian populations, with most evidence originating from China. The included studies covered three broad clinical targets: gastric cancer, precancerous gastric lesions or high-risk gastric screening, and related gastrointestinal disorders. Study designs ranged from single-center case-control studies to multicenter prospective cohorts. Sample sizes varied substantially, from small exploratory cohorts of fewer than 200 participants to large multicenter datasets involving more than 3,000 participants. Reference standards were most robust in studies that used gastroscopy and histopathology, particularly for gastric cancer and precancerous gastric lesions, whereas some studies of related gastrointestinal disorders relied on clinically defined diagnostic categories.

**Table 1:** Clinical and design characteristics of the included studies.

Study	Country/setting	Design and centers	Sample and groups	Target condition	Evidence category	Reference standard/diagnostic label
Zhu et al. (2022) (31)	China; Shanxi	Development/case-control study; 2 centers	703 tongue images: 103 GC and 600 non-GC controls; augmented to 4,375 images	Gastric cancer	Primary gastric evidence	Unclear; diagnosis by specialized physicians, histopathological confirmation not clearly reported
Yuan et al. (2023) (30)	China	Prospective multicenter cohort; 17 centers	3,663 participants; development from 10 centers and external validation from 7 centers	Gastric cancer	Primary gastric evidence	Gastroscopy and histopathology
Qiao et al. (2024) (34)	China; Qilu Hospital	Single-center diagnostic-model study	949 patients and 2,167 images; heterogeneous diseased and healthy-control groups	Mixed GI diseases including GC, H. pylori infection, bile reflux, and esophagitis	Primary gastric evidence	Mixed: endoscopy, biopsy/histology, and disease-specific diagnostic tests
Ma et al. (2023) (27)	China	Development and external-validation study; 2 centers	325 in development cohort, 1,995 in external-validation cohort, and 95 in follow-up cohort	Precancerous gastric lesions	Primary gastric evidence	Gastroscopy and histopathology
Wang et al. (2024) (28)	China; Fujian and China-Japan Friendship Hospital	Development and external-validation study; 2 centers	1,034 in development cohort and 143 in external-validation cohort	PLGC and high-risk PLGC	Primary gastric evidence	EGD and biopsy/histopathology
Yu et al. (2025) (29)	China	Multicenter cohort; 9 hospitals	2,229 participants: 1,006 PLGC, 818 GC, and 405 healthy controls	PLGC, GC, and healthy controls	Primary gastric evidence	Histopathological confirmation
Wu et al. (2020) (32)	Taiwan	Single-center case-control study	134 participants: 67 GERD and 67 healthy controls	Gastroesophageal reflux disease	Contextual	Endoscopic confirmation
Dai et al. (2024) (33)	China	Single-center cross-sectional study	166 participants: 92 MAFLD and 74 healthy controls	Metabolic-associated fatty liver disease	Contextual	Clinically defined using metabolic criteria with imaging/biomarker or histological evidence
Duan et al. (2018) (26)	China; Ci County high-risk screening setting	Population screening study	3,053 participants: 79 EC, 134 premalignant lesions, and 2,840 normal controls	Esophageal cancer and premalignant lesions	Contextual	Histopathological confirmation

EC, esophageal cancer; GC, gastric cancer; GERD, gastroesophageal reflux disease; GI, gastrointestinal; H. pylori, Helicobacter pylori; MAFLD, metabolic-associated fatty liver disease; non-GC, non-gastric cancer; PLGC, precancerous gastric lesion(s).

Reference-standard quality varied across the included studies. Histopathological confirmation was reported for the principal gastric cancer or precancerous-lesion studies by Yuan et al., Ma et al., Wang et al., and Yu et al., as well as the esophageal cancer study by Duan et al. Other studies used endoscopic confirmation, clinically defined diagnostic criteria, or mixed diagnostic evidence (26–30). For Zhu et al., the report stated that participants had been definitively diagnosed by specialized physicians, but did not provide sufficient information to establish histopathological confirmation (31). Accordingly, diagnostic performance estimates were interpreted in the context of the reference standard used rather than being considered directly interchangeable across studies.

Across studies, tongue images were acquired using either dedicated tongue-imaging systems or more pragmatic camera-based approaches, including smartphone photography. AI architectures included traditional machine-learning classifiers based on handcrafted color, texture, and geometric features; convolutional neural networks; transformer-based models; segmentation-assisted pipelines; and multimodal models combining tongue images with clinical, questionnaire, biomarker, or microbiome data. External validation was performed in only a minority of studies, and calibration assessment was rarely reported.

### 3.3 Image acquisition, preprocessing, and segmentation

Image acquisition varied considerably across studies, reflecting the absence of a standardized tongue-imaging protocol in this field. Some studies used dedicated tongue-imaging instruments designed to reduce variability in illumination, camera distance, and patient positioning. Duan et al. used the DS01-B computerized tongue acquisition system in a large screening population for esophageal cancer and premalignant lesions (26). Wu et al. used the Automatic Tongue Diagnosis System for GERD assessment (32), while Dai et al. used the TFDA-1 tongue diagnosis instrument for MAFLD classification (33). Zhu et al. used a bespoke tongue-image capture system with high-resolution image acquisition for gastric cancer classification (31). These instrument-based approaches are more suitable for controlled research settings, but their generalizability to community-based or smartphone-based screening remains uncertain.

Other studies adopted more pragmatic acquisition strategies. Qiao et al. used smartphone-based tongue photography under more variable conditions, which increases potential deployability but also introduces heterogeneity in lighting, camera sensor characteristics, angle, and image quality (34). This distinction between controlled acquisition and real-world imaging is important because tongue color, coating thickness, moisture, and texture are central diagnostic features, and all of these may be influenced by device and lighting variability. Across the included studies, details regarding color calibration, image-quality exclusion criteria, and reproducibility of image acquisition were inconsistently reported.

Segmentation and preprocessing strategies also differed across studies. Several deep-learning studies used automated segmentation to isolate the tongue from surrounding facial structures before classification. Zhu et al. used DeepLabv3 + as part of a segmentation-assisted framework before EfficientNet-B0 classification (31). Qiao et al. used U-Net for automatic tongue segmentation before feature fusion and classification (34). Ma et al. used YOLOv5 for tongue localization and segmentation in the AITongue framework (27), and Wang et al. used the Segment Anything Model as part of the AITonguequiry pipeline (28). Dai et al. applied Tongue-GAN for segmentation before combining tongue-image and microbiome features (33). These approaches demonstrate that segmentation has become a central step in modern tongue-image AI pipelines, although few studies formally evaluated how segmentation error affected downstream diagnostic performance.

Data augmentation and class-imbalance handling were reported in five of the nine studies (55.6%). Explicit patient-level separation between development and testing datasets was described in six studies (66.7%). However, only two studies (22.2%) explicitly stated that augmentation was performed after patient-level splitting. Seven studies (77.8%) were judged to have low concern for data leakage based on the available reporting, whereas two studies (22.2%) had possible leakage risk because the splitting procedure was described at the image level or did not provide sufficient information to establish participant-level separation. These findings should not be interpreted as proof that leakage occurred in the latter studies; rather, they indicate that leakage could not be confidently excluded. This distinction is important because augmented images derived from the same participant may otherwise cross training and testing partitions and produce optimistically biased estimates.

Figure 2 shows an overview of the language image analysis pipeline.

**Figure 2:**
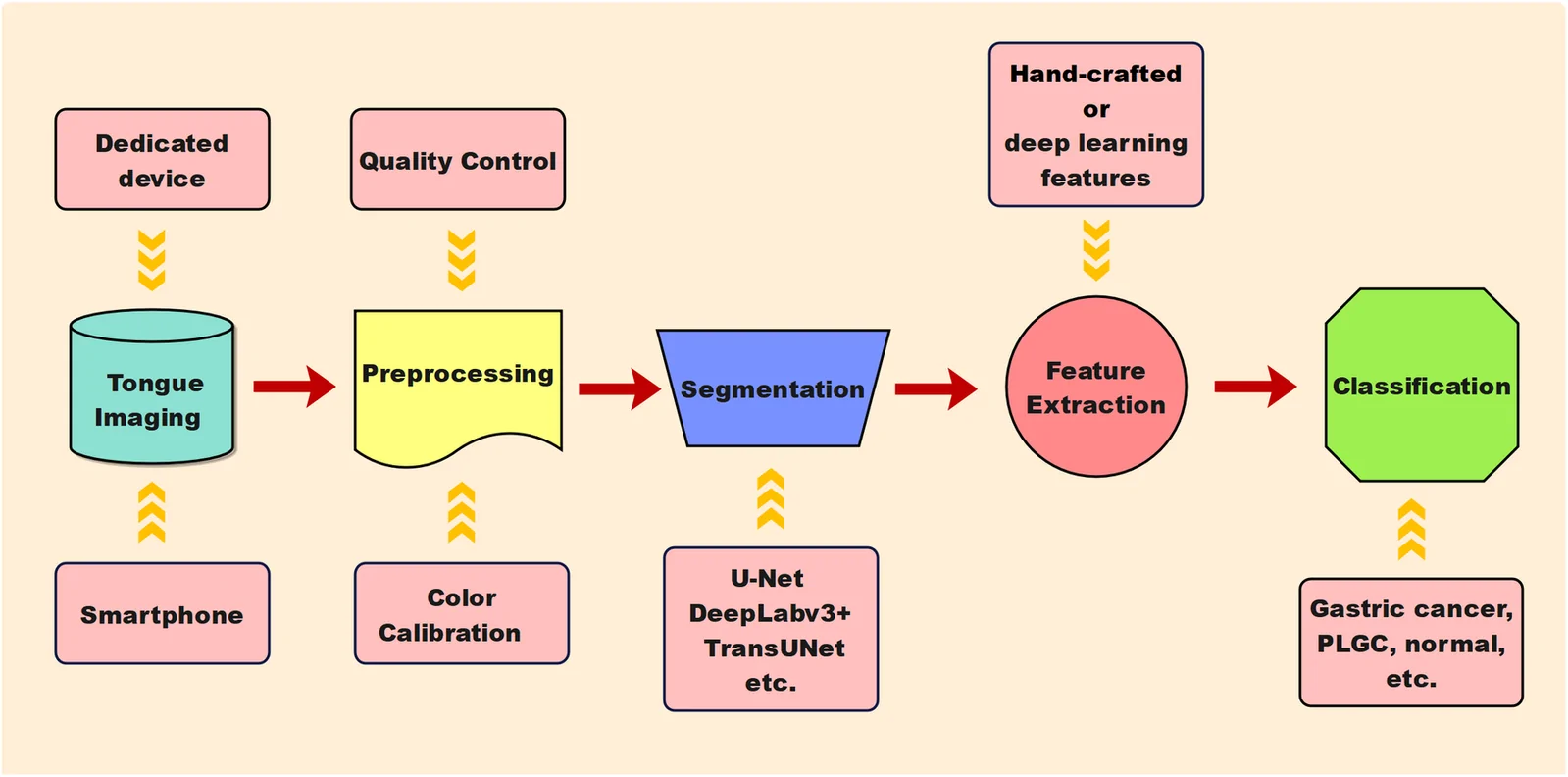
Workflow of AI-based tongue-image analysis. Overview of the tongue-image analysis pipeline, from image acquisition and image-quality control to preprocessing, tongue segmentation, feature extraction, model development, validation, and AI-based classification or risk stratification for gastric cancer, precancerous gastric lesions, and related gastrointestinal disorders. AI, artificial intelligence; CNN, convolutional neural network; ML, machine learning.

### 3.4 AI model architectures and multimodal strategies

The included studies used a broad range of AI architectures, reflecting the rapid methodological evolution of tongue-image analysis. (Table 2) Traditional machine-learning approaches relied on handcrafted color, texture, geometric, and coating-related features. Duan et al. used logistic regression with tongue color-space features and demographic variables for esophageal cancer screening (26), while Wu et al. used logistic regression to evaluate tongue indices associated with GERD (32). Dai et al. compared several machine-learning classifiers and found that XGBoost performed best when tongue-image features were combined with microbiome and clinical data (33). These approaches offer relatively transparent feature interpretation, but they depend heavily on predefined features and may fail to capture complex visual patterns.

**Table 2:** Model categories used in tongue-image AI for gastric cancer and related gastrointestinal disorders.

Category	Representative architectures	Example studies	Advantages	Disadvantages	Interpretability	Data requirement
Traditional ML	Logistic regression, SVM, RF, XGBoost	Duan et al. (26), Wu et al. (32), Dai et al. (33)	Low computational cost; transparent feature importance; works with small datasets	Limited capacity for complex nonlinear patterns; requires handcrafted feature engineering	High	Low
CNN-based	ResNet50, EfficientNet-B0, Faster R-CNN	Zhu et al. (31), Ma et al. (27)	High accuracy; automatic feature learning; transfer learning from ImageNet; well-established tooling	Requires large labeled datasets; limited interpretability without *post-hoc* methods; sensitive to domain shift	Low–Medium	High
Transformer-based	Swin Transformer, ViT	Yu et al. (29), Yuan et al. (30)	Global attention modeling; captures long-range dependencies; competitive with or exceeds CNNs on image tasks	Very high data and compute requirements; even less interpretable than CNNs; limited pretrained models for medical imaging	Low	Very High
Segmentation-assisted	DeepLabv3+ + classifier, U-Net + classifier	Zhu et al. (31), Qiao et al. (34), Yuan et al. (30)	Joint optimization of segmentation and classification; focuses model on tongue region; reduces background noise	Increased training complexity; segmentation quality directly affects classification; two-stage pipeline may propagate errors	Medium	High
Multimodal	Image + clinical variables, Image + microbiome	Wang et al. (28), Yuan et al. (30), Dai et al. (33), Yu et al. (29)	Richer feature representation; may capture complementary information beyond visual appearance; potential for higher accuracy	Increased data collection burden; missing modality handling; risk of overfitting with many input features; harder to standardize	Medium	High

AI, artificial intelligence; CNN, convolutional neural network; ML, machine learning; RF, random forest; SAM, Segment Anything Model; SVM, support vector machine; TCM, traditional Chinese medicine; TransFG, transformer-based fine-grained model; ViT, vision transformer; XGBoost, extreme gradient boosting.

Convolutional neural networks were the most common deep-learning approach. Zhu et al. used EfficientNet-B0 after tongue segmentation to classify gastric cancer, reporting high internal performance (31). Ma et al. developed AITongue, a ResNet50-based model integrated with clinical risk factors for screening precancerous gastric lesions (27). Wang et al. also used a ResNet-based image encoder within a multimodal framework that combined tongue images with inquiry data (28). CNN-based models allow automatic feature learning from images, but their interpretability remains limited and their performance may be sensitive to domain shift between acquisition devices, centers, and populations.

Transformer-based and segmentation-assisted models appeared in more recent studies. Yuan et al. compared APINet, TransFG, and DeepLabV3 + across a large multicenter gastric cancer cohort and showed that multiple architectures achieved strong discrimination, particularly when tongue images were combined with blood biomarkers (30). Yu et al. used a Swin Transformer for tongue-image classification and combined this with machine-learning analysis of questionnaire and TCM constitution data in a multicenter cohort of PLGC, GC, and healthy participants (29). These studies suggest that transformer-based models and multimodal fusion may improve representation learning, but their data requirements are high and external validation remains limited.

Multimodal modeling was a recurring theme in the stronger or more recent studies. Ma et al. combined tongue images with age, sex, and H. pylori status (27). Wang et al. combined tongue images with demographic, behavioral, and symptom-related inquiry information through a multi-head attention fusion mechanism (28). Yuan et al. combined tongue images with blood biomarkers in a multicenter gastric cancer cohort (30), and Dai et al. integrated tongue images with tongue-coating and intestinal microbiome data (33). These studies suggest that tongue images may be more clinically useful when incorporated into broader risk-prediction frameworks rather than used as isolated diagnostic tests. However, the incremental value of each modality was not consistently quantified, and multimodal models may be more difficult to standardize for real-world screening.

Five of the nine included studies used multimodal inputs; four directly permitted comparison of multimodal performance with image-only and clinical/biomarker-only approaches (Yuan et al., Ma et al., Wang et al., and Dai et al.). In all four studies, the multimodal model achieved a numerically higher AUC than at least one relevant single-modality comparator. However, the strength of evidence for incremental discrimination varied. Ma et al. reported an external AUC of 0.75 for the multimodal model compared with 0.68 for the clinical-risk-factor model (*p* < 0.01), while Wang et al. reported validation-set AUCs of 0.74 for the combined model, 0.70 for image-only, and 0.66 for inquiry-only modelling, with DeLong testing indicating statistically significant differences (*p* < 0.05). Yuan et al. and Dai et al. also reported higher AUCs for combined models, but formal statistical testing of the incremental contribution of tongue imaging was not clearly reported. Thus, the available evidence supports a potential incremental contribution of tongue-image information but does not establish that multimodal AUC gains are consistently statistically significant or clinically meaningful (27, 28, 30, 33).

### 3.5 Evidence by target condition

#### 3.5.1 Gastric cancer

The strongest direct evidence for gastric cancer came from studies that used deep-learning models on tongue images, either alone or with additional clinical or biomarker data. Zhu et al. developed a segmentation-assisted framework combining DeepLabv3+, handcrafted tongue features, and EfficientNet-B0 for gastric cancer classification (31). The study included 703 tongue images from two centers in China, comprising 103 gastric cancer cases and 600 non-gastric-cancer controls. After data augmentation, the model achieved high internal performance, with reported accuracy of 93.6%, sensitivity of 92.6%, and F1-score of 92.6%. Feature-importance analysis identified dry saliva, fat tongue, and white tongue as discriminative features. Although the study integrated TCM-domain knowledge into the feature-engineering process, its case-control design, relatively small number of gastric cancer cases, and lack of external validation limit the generalizability of the findings.

Yuan et al. provided the most methodologically robust gastric cancer evidence among the included studies (30). This prospective multicenter cohort enrolled 3,663 participants across 17 centers in China. Ten centers contributed to model development, and seven centers were used for external validation. All diagnoses were confirmed by gastroscopy and pathology. The study compared APINet, TransFG, and DeepLabV3 + models using multimodal inputs that combined tongue images with blood biomarkers. In internal validation, AUC values ranged from 0.88 to 0.92, with sensitivity between 0.83 and 0.88 and accuracy between 0.81 and 0.83. In external validation, AUC decreased to 0.83–0.88 and accuracy to 0.75–0.77. Although this decline indicates some performance loss under external validation, the study remains the strongest evidence supporting the potential use of tongue-image AI for gastric cancer screening because of its prospective design, multicenter recruitment, histopathological verification, and external validation.

Qiao et al. evaluated a hybrid intelligent tongue diagnosis model in a broader gastrointestinal disease cohort that included gastric cancer, H. pylori infection, bile reflux, and esophagitis (34). The study used smartphone-based image acquisition and a hybrid architecture combining handcrafted features with deep visual features extracted through SE-ResNet50 and slot attention. The model achieved an AUC of 0.886, sensitivity of 0.965, accuracy of 0.849, and F1-score of 0.902 in internal validation. Grad-CAM visualization suggested that the middle region of the tongue was particularly relevant for stomach-related conditions. However, because gastric cancer was grouped with other gastrointestinal diseases, the model's disease-specific performance for gastric cancer cannot be clearly separated. The absence of external validation and the heterogeneous disease grouping limit the specificity of the conclusions for gastric cancer screening.

#### 3.5.2 Precancerous gastric lesions and high-risk screening

Three studies evaluated tongue-image AI for precancerous gastric lesions or high-risk gastric screening, a clinically distinct task from established gastric cancer detection. Unlike binary gastric cancer classification, PLGC screening requires identification of earlier pathological stages such as atrophic gastritis, intestinal metaplasia, and dysplasia, which may produce subtler image signals and greater diagnostic overlap with non-PLGC conditions.

Ma et al. developed AITongue, a ResNet50-based model for PLGC screening using tongue images and clinical risk factors (27). The development cohort included 325 participants, comprising 55 PLGC cases and 270 non-PLGC controls, while the external validation cohort included 1,995 participants. The image-only model achieved an AUC of 0.75 and accuracy of 0.69 in internal validation. In external validation, AUC remained 0.75, although accuracy decreased to 0.64. A follow-up cohort of 95 patients yielded an AUC of 0.71 for PLGC risk prediction. Tongue Diagnostic Label correlation analysis identified greasy, fissured, dark, yellow, and thick coating features as being most strongly associated with PLGC. The inclusion of external validation is an important strength, but the moderate performance indicates that PLGC classification is more difficult than established gastric cancer classification.

Wang et al. extended this approach by developing AITonguequiry, a multimodal model combining tongue images with inquiry information such as demographics, behaviors, and physical symptoms (28). The development cohort included 1,034 participants from Fujian, and an external validation cohort included 143 participants from the China-Japan Friendship Hospital. The model achieved an internal AUC of 0.74 for PLGC and 0.82 for high-risk PLGC. In external validation, AUC decreased to 0.69 for PLGC and 0.76–0.77 for high-risk PLGC. The model's attention mechanism highlighted tongue coating color, coating thickness, moisture, and tongue body shape as predictive features. The observed decline in external validation again suggests that models trained on tongue images may be sensitive to population and acquisition differences.

Yu et al. evaluated a multimodal approach for classifying PLGC, gastric cancer, and healthy participants in a cohort of 2,229 individuals from nine hospitals in China (29). A Swin Transformer was used for image-based analysis, while random forest was selected for questionnaire and TCM constitution data. The Swin Transformer achieved 73.33% accuracy, and the random forest model achieved 85.65% accuracy, with AUC values above 0.8 across disease categories. SHAP analysis identified age, TCM constitution, tongue color, irregular meals, and greasy fur as important predictors. Although the multicenter design is a strength, the absence of dedicated external validation for the image-based component limits conclusions about generalizability.

Overall, PLGC studies showed lower and less stable performance than gastric cancer studies. AUC values for PLGC or high-risk PLGC generally ranged from approximately 0.69 to 0.82, whereas the strongest gastric cancer models reported AUC values of 0.83–0.92 under external validation and higher values under internal validation. Across PLGC studies, coating-related features, particularly greasy, thick, yellow, or abnormal coating patterns, were repeatedly identified as relevant predictors. Multimodal models appeared more informative than image-only models, but the available evidence is insufficient to determine whether tongue images provide independent clinical value beyond established risk factors.

#### 3.5.3 Contextual evidence from other gastrointestinal disorders

Three included studies evaluated gastrointestinal conditions outside gastric cancer or precancerous gastric lesions and are therefore considered contextual rather than primary evidence for the review’s gastric-cancer question.

Wu et al. evaluated tongue diagnosis indices for GERD in a case-control study of 134 participants, including 67 patients with GERD and 67 healthy controls (32). The study used the Automatic Tongue Diagnosis System and logistic regression across nine tongue features. Saliva amount and tongue fur in the spleen-stomach area were significantly associated with GERD, but discrimination was modest, with AUC values of 0.61–0.62. This study supports the possibility that specific tongue features correlate with upper gastrointestinal disorders, but the small sample size and limited performance restrict its clinical implications.

Dai et al. applied tongue-image AI combined with microbiome analysis for MAFLD diagnosis in a cross-sectional study of 166 participants, including 92 MAFLD patients and 74 healthy controls (33). The best-performing model was XGBoost, which combined tongue-image features, tongue-coating and intestinal microbiome data, and clinical variables such as age, sex, and BMI. The model achieved an AUC of 0.99, sensitivity of 94.57%, specificity of 98.65%, accuracy of 96.39%, and F1-score of 0.97. The study also reported Hosmer-Lemeshow calibration assessment, which was uncommon among the included studies. However, the small sample size, single-center design, and baseline differences in age and BMI between groups raise concerns about overfitting and confounding.

Duan et al. evaluated computer-aided tongue inspection for preliminary screening of esophageal cancer and premalignant lesions in 3,053 participants from a high-risk population (26). The model used logistic regression with tongue color-space features, age, and sex, and achieved sensitivity of 61.6%, specificity of 72.8%, and accuracy of 72.3%. Although the target condition was esophageal rather than gastric cancer, this study is relevant because it represents one of the largest real-world tongue-image screening studies and demonstrates the feasibility of population-level deployment using standardized tongue-image acquisition. Nevertheless, the diagnostic performance was modest and the model was not externally validated.

These studies demonstrate that tongue-image AI has been investigated across a broader gastrointestinal disease spectrum; however, differences in target organ, disease definition, reference standard, and clinical application prevent their performance estimates from being directly compared with gastric cancer studies. These findings provide biological and methodological context for gastrointestinal applications of tongue-image AI but should not be interpreted as evidence of diagnostic accuracy for gastric cancer.

### 3.6 Diagnostic performance, validation, and interpretability

Diagnostic performance varied substantially across studies, reflecting differences in disease target, study design, sample size, acquisition protocol, and validation strategy. Table 3 summarizes the main performance metrics reported by the included studies. Internally validated models reported AUC values ranging from 0.61 to 0.99 and accuracy values ranging from 61.6% to 96.4%. The highest internal performance was observed in studies using deep-learning or multimodal approaches, particularly for gastric cancer and MAFLD. However, high internal performance should be interpreted cautiously because several studies used single-center datasets, case-control sampling, small disease groups, or limited reporting of patient-level data splitting.

**Table 3:** Comparative performance of all 9 tongue-image AI studies reviewed for gastric cancer, precancerous lesions, and other gastrointestinal disorders.

Study	Input data and model	Validation strategy	Main reported performance	Interpretability/key image signal
*Gastric cancer studies*				
Zhu et al. (2022) (31)	Tongue images with DeepLabv3 + segmentation, handcrafted TCM-informed features, and EfficientNet-B0	Internal train/validation/test split (8:1:1); no external validation	Accuracy 93.6%; sensitivity 92.6%; F1-score 92.6%; 95% CIs not reported.	XGBT feature importance; dry saliva, fat tongue, and white tongue
Yuan et al. (2023) (30)	Tongue images plus blood biomarkers; APINet, TransFG, and DeepLabV3 + models	Internal validation and external validation across 7 independent centers	Internal AUC 0.88–0.92; external AUC 0.83–0.88, with external AUCs of 0.85 (95% CI 0.83–0.88), 0.88 (95% CI 0.86–0.91), and 0.83 (95% CI 0.80–0.86) for APINet, TransFG, and DeepLabV3+, respectively; external accuracy 0.75–0.77.	Activation maps, attention patches, and segmentation pixels; coating thickness and moisture
Qiao et al. (2024) (34)	Smartphone tongue images; U-Net segmentation; SE-ResNet50 with slot attention plus stacked ML ensemble	Internal cross-validation; no external validation	AUC 0.886; sensitivity 0.965; accuracy 0.849; F1-score 0.902; 95% CIs not reported.	Grad-CAM; middle tongue region for stomach-related conditions
*Precancerous lesion studies*				
Ma et al. (2023) (27)	AITongue: ResNet50 image model combined with age, sex, and H. pylori status	5-fold cross-validation, independent external validation, and follow-up cohort	Internal AUC 0.75 and accuracy 0.69; external AUC 0.75 and accuracy 0.64; follow-up AUC 0.71; 95% CIs not reported.	TDL correlation; greasy, fissured, dark, yellow, and thick coating
Wang et al. (2024) (28)	AITonguequiry: ResNet image encoder plus inquiry-data encoder with multi-head attention fusion	Internal split and independent external validation	PLGC AUC 0.74 (95% CI 0.71–0.76) internally and 0.69 externally; high-risk PLGC AUC 0.82 internally and 0.76–0.77 externally; internal sensitivity 73.33% (95% CI 71.59–75.04) and specificity 66.67% (95% CI 63.94–69.43).	Attention-based features; coating color, thickness, moisture, and tongue body shape
Yu et al. (2025) (29)	Swin Transformer for tongue images plus RF model for questionnaire and TCM constitution data	Internal testing; no dedicated external validation for the image model	Swin Transformer accuracy 73.33%; RF accuracy 85.65%; AUC >0.80 across categories; validation-set nomogram AUC 0.876 (95% CI 0.848–0.905); 95% CI for the Swin Transformer performance not reported.	SHAP and heatmaps; age, TCM constitution, tongue color, irregular meals, and greasy fur
*Contextual evidence from related gastrointestinal disorders*				
Wu et al. (2020) (32)	ATDS-derived tongue indices evaluated with logistic regression	No explicit train/test split; no external validation	AUC 0.606 (95% CI 0.510–0.702) for saliva amount and 0.615 (95% CI 0.518–0.713) for tongue-fur percentage in the spleen–stomach area.	Odds ratios and *p*-values; saliva amount and tongue fur in spleen-stomach area
Dai et al. (2024) (33)	TFDA-1 images with Tongue-GAN segmentation, microbiome features, clinical variables, and XGBoost	5-fold cross-validation; no external validation	AUC 0.99; sensitivity 94.57%; specificity 98.65%; accuracy 96.39%; F1-score 0.97; 95% CIs not reported.	LEfSe and feature importance; Streptococcus, Rothia, and tongue color features
Duan et al. (2018) (26)	DS01-B tongue color-space features combined with age and sex in logistic regression	Hold-out testing; no external validation	Sensitivity 61.6%; specificity 72.8%; accuracy 72.3%; 95% CIs not reported.	Regression coefficients; saturation, b-channel, prickle, ecchymosis, red/dark-red tongue

Acc, accuracy; APINet, attention pyramid integrated network; ATDS, Automatic Tongue Diagnosis System; AUC, area under the receiver operating characteristic curve; F1, F1-score; GC, gastric cancer; Grad-CAM, gradient-weighted class activation mapping; H. pylori, Helicobacter pylori; LEfSe, linear discriminant analysis effect size; ML, machine learning; PLGC, precancerous gastric lesion(s); RF, random forest; Sens, sensitivity; Spec, specificity; TCM, traditional Chinese medicine; TDL, Tongue Diagnostic Label; TransFG, transformer-based fine-grained model; XGBT/XGBoost, extreme gradient boosting.

External validation was performed in only three studies. Yuan et al. externally validated gastric cancer models across seven independent centers and observed AUC decreases from 0.88–0.92 internally to 0.83–0.88 externally (30). Ma et al. reported stable AUC but lower accuracy in external validation of PLGC screening, with accuracy decreasing from 0.69 to 0.64 (27). Wang et al. observed a decline in PLGC AUC from 0.74 internally to 0.69 externally, with high-risk PLGC AUC decreasing to approximately 0.76–0.77 (28). These results indicate that external validation tends to reduce apparent performance, particularly for PLGC, and that internally validated estimates may overstate clinical readiness.

Interpretability was addressed in most but not all studies. Traditional models provided relatively direct interpretability through regression coefficients, odds ratios, feature importance, or statistical associations, as seen in Duan et al., Wu et al., and Dai et al. (26, 32, 33). Deep-learning studies used *post-hoc* or attention-based methods, including Grad-CAM, heatmaps, gate activation maps, multi-head attention visualization, SHAP, and Tongue Diagnostic Label correlation analysis (27–31, 34). Across these methods, coating-related features repeatedly emerged as important, including coating thickness, coating color, greasiness, moisture, and tongue body shape. This convergence across different architectures and disease targets suggests that tongue coating may be one of the most consistent visual signals in gastric and gastrointestinal tongue-image AI. However, most interpretability analyses were descriptive and did not establish causal or mechanistic links between model attention and underlying pathology.

### 3.7 Risk of bias, calibration, and reporting quality

Risk of bias varied across the included studies, particularly in the analysis domain. Two studies (Zhu et al. and Dai et al.) were judged to have high risk in the analysis domain because participant-level separation could not be established and/or image-level splitting created potential leakage concerns (31, 33). The remaining seven studies were judged to have low risk in the analysis domain, although several had incomplete reporting of augmentation procedures, calibration, sample-size justification, or external validation. Overall applicability concerns were high for four studies (Zhu et al., Qiao et al., Wu et al., and Dai et al.), primarily because of case-control or highly selected populations, related gastrointestinal rather than gastric cancer/precancerous gastric lesion targets, specialized image-acquisition conditions, or multimodal inputs that are not representative of simple pre-endoscopic screening (31–34). The remaining five studies had low applicability concerns.

Unclear judgments primarily reflected incomplete reporting rather than evidence that the relevant methodological procedure was inappropriate. For example, Zhu et al. did not report whether the 8:1:1 image split was performed at the participant level or whether augmentation preceded or followed partitioning, while Yu et al. did not clearly specify the timing of augmentation relative to the 70:30 split (29, 31). These studies were therefore not assumed to have experienced leakage solely because the procedures were incompletely reported.

Calibration reporting was limited across the evidence base. Yuan et al. provided probability-distribution plots, Yu et al. presented a visual calibration plot, and Dai et al. reported the Hosmer–Lemeshow test; the remaining studies did not report formal calibration assessment (29, 30, 33). External validation was reported by Yuan et al., Ma et al., Wang et al., and Yu et al., whereas the other studies lacked independent external validation (27–30, 33). Detailed domain-level judgments, signaling questions, and study-specific reasons for unclear or high-risk judgments are provided in Table 4 and Supplementary Table S5.

**Table 4:** Risk of bias, applicability, analysis, and calibration reporting across the included studies.

Study	Participant selection	Index test	Reference standard	Flow and timing	Analysis	Applicability	Calibration reporting	Key concerns
Zhu et al. (2022) (31)	High risk	Low risk	Low risk	Unclear risk	High risk	High concern	None reported	Case-control design with only 103 original GC images; participant-level separation and timing of augmentation were not reported, creating possible leakage risk; no external validation or calibration assessment; bespoke image-acquisition system limits real-world applicability.
Yuan et al. (2023) (30)	Low risk	Low risk	Low risk	Unclear risk	Low risk	Low concern	Probability-distribution plots only	Large prospective multicenter study with independent geographic external validation and histopathological confirmation; however, formal calibration assessment was limited and model-development details were incompletely reported.
Qiao et al. (2024) (34)	Low risk	Low risk	Low risk	Unclear risk	Low risk	High concern	None reported	Participant-level separation and training-only resampling/augmentation were reported, but the study targeted heterogeneous related gastrointestinal disorders rather than GC/PLGC; no external validation or calibration assessment.
Ma et al. (2023) (27)	High risk	Low risk	Low risk	Low risk	Low risk	Low concern	None reported	Small development cohort with only 55 PLGC cases and no reported sample-size calculation; external validation was performed, but calibration was not assessed.
Wang et al. (2024) (28)	Low risk	Low risk	Low risk	Low risk	Low risk	Low concern	None reported	Patient-level separation and external validation were reported, with augmentation performed during training; external performance was lower than internal performance and calibration was not reported.
Yu et al. (2025) (29)	High risk	Low risk	Low risk	Unclear risk	Low risk	Low concern	Visual calibration plot only	Case-control elements and deliberate recruitment of healthy participants introduce selection concerns; augmentation timing was unclear and participant-level exclusion from both partitions was not explicitly documented; no dedicated external validation of the image model and limited calibration assessment.
Wu et al. (2020) (32)	Low risk	Low risk	Low risk	Unclear risk	Low risk	High concern	None reported	Small single-center GERD study using statistical associations rather than an independently validated predictive model; no external validation or calibration assessment; target condition is related GI disease rather than GC/PLGC and image acquisition required specialized ATDS conditions.
Dai et al. (2024) (33)	High risk	Low risk	Low risk	Unclear risk	High risk	High concern	Hosmer-Lemeshow test	Image-level splitting was reported for the GAN component without establishing participant-level separation; small cross-sectional MAFLD cohort, substantial selection/confounding concerns, no external validation, and multimodal inputs included microbiome sequencing that is not representative of simple pre-endoscopic screening.
Duan et al. (2018) (26)	Low risk	Low risk	Low risk	Unclear risk	Low risk	High concern	None reported	Large screening-based cohort with prospective histological confirmation and participant-level training/test grouping, but no external validation or calibration assessment; target condition was esophageal cancer/premalignant lesions rather than GC/PLGC.

ECE, expected calibration error; GC, gastric cancer; MAFLD, metabolic-associated fatty liver disease; PLGC, precancerous gastric lesions.

## 4 Discussion

### 4.1 Principal findings

This systematic review synthesized the available evidence on artificial intelligence-based tongue-image analysis for non-invasive screening of gastric cancer, precancerous gastric lesions, and related gastrointestinal disorders. Across 9 included studies, the field was characterized by rapid methodological development, heterogeneous study designs, and an evidence base that remains promising but not yet clinically mature. The strongest evidence was observed for gastric cancer detection, particularly in multicenter cohorts with histopathological confirmation and external validation. In contrast, evidence for precancerous gastric lesions was more preliminary, with lower and less stable performance, and evidence for other gastrointestinal disorders remained exploratory.

Several consistent findings emerged across the reviewed studies. First, tongue-image AI models were able to extract diagnostically meaningful visual information from tongue photographs, especially coating-related features such as coating thickness, color, greasiness, moisture, and tongue body shape. Second, externally validated models generally showed lower performance than internally validated models, suggesting that single-center internal validation may overestimate clinical performance. Third, multimodal models that combined tongue images with clinical variables, questionnaire data, blood biomarkers, or microbiome data tended to perform better than image-only approaches, although the independent incremental value of tongue images was not consistently quantified. Fourth, calibration reporting was largely absent, despite being essential for any model intended to guide referral decisions in a screening or triage setting.

### 4.2 Interpretation of findings

The reviewed evidence suggests that tongue-image AI may be best understood as an emerging risk-stratification technology rather than a stand-alone diagnostic test. Gastric cancer models, particularly those evaluated in multicenter cohorts, achieved moderate to high discrimination, and the externally validated results from Yuan et al. provide the most convincing evidence that tongue-image features can retain predictive value beyond a single development dataset (35). However, even in this stronger study, performance decreased under external validation, indicating that acquisition setting, patient population, and center-specific factors can influence model behavior. This finding is important because a screening tool must perform reliably across clinical environments rather than only under development conditions.

The lower performance observed in PLGC studies is clinically plausible. Precancerous gastric lesions represent a biologically and morphologically heterogeneous group that includes atrophic gastritis, intestinal metaplasia, and dysplasia. These conditions may produce subtler systemic or oral manifestations than established gastric cancer and may overlap with benign inflammatory or functional gastrointestinal disorders. The moderate AUC values reported by Ma et al. and Wang et al., together with performance decline in external validation, suggest that tongue images alone may be insufficient for robust PLGC classification (27, 28). The stronger performance of multimodal approaches in this setting indicates that tongue-image features may be more useful when embedded within a broader clinical risk model.

The repeated identification of coating-related features across different models and target diseases is one of the most biologically coherent findings of this review. Traditional Chinese medicine has long emphasized tongue coating as a marker of digestive function, and modern studies of tongue-coating microbiota and metaproteomics provide a plausible biological substrate for this association (14–22). However, the current evidence remains associative. None of the included AI studies established a mechanistic link between specific image features and gastric pathology, and most interpretability methods remained descriptive. Grad-CAM, attention maps, SHAP, and feature-importance analyses can identify model-relevant regions or variables, but they do not prove that the model is using pathophysiologically meaningful signals rather than confounded image patterns. (Figure 3).

**Figure 3:**
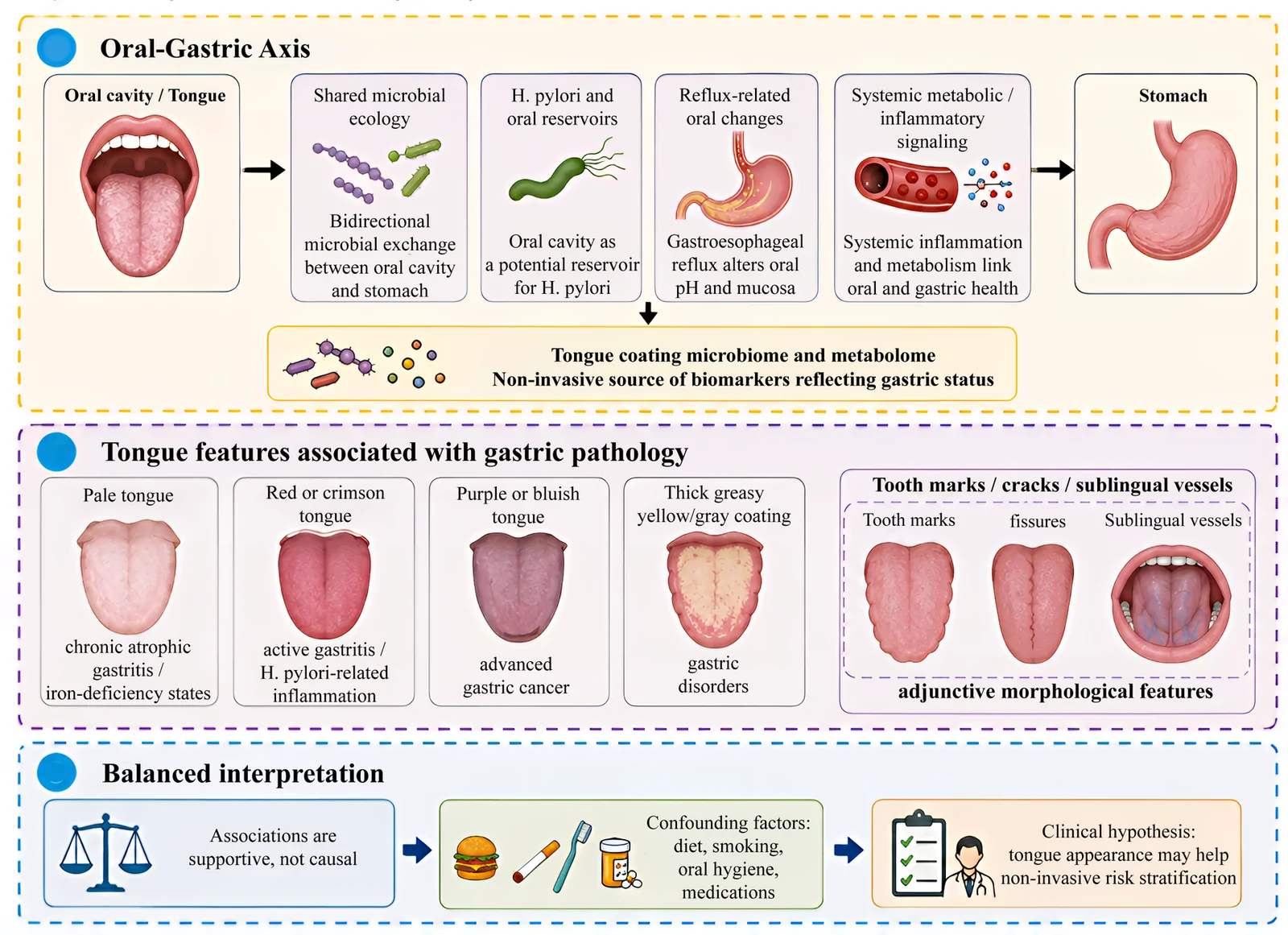
Biological rationale and tongue-image features relevant to gastric disease. Conceptual summary of the proposed biological and diagnostic rationale linking tongue appearance with gastric and upper gastrointestinal pathology. The figure highlights the oral-gastric axis, tongue-coating microbiome and metabolomic alterations, and frequently reported tongue-image features such as coating thickness, coating color, greasiness, moisture, and tongue body morphology. GC, gastric cancer; GI, gastrointestinal; PLGC, precancerous gastric lesion(s); TCM, traditional Chinese medicine.

Overall, the certainty of evidence was low for both gastric cancer and precancerous gastric lesion/high-risk screening applications, while the certainty of contextual evidence from other gastrointestinal disorders was very low. The principal limitations were study-level risk of bias, heterogeneous reference standards and populations, incomplete reporting of confidence intervals and calibration, and limited independent external validation. Although some individual studies—particularly the prospective multicenter gastric cancer study by Yuan et al.—provided comparatively stronger evidence, the evidence base as a whole remains insufficient to establish reliable clinical effectiveness or generalizability (30). Accordingly, tongue-image AI should currently be considered a promising adjunctive pre-endoscopic triage approach rather than a clinically established screening or diagnostic tool.

### 4.3 Clinical implications

The most realistic near-term clinical role for tongue-image AI is as a low-cost, non-invasive pre-endoscopic triage tool. Such a tool would not replace gastroscopy or histopathology, but could help identify individuals who may benefit most from confirmatory endoscopy. This positioning is important because the clinical question is not whether tongue-image AI can diagnose gastric cancer definitively, but whether it can safely enrich the screened population for individuals at higher risk while reducing unnecessary endoscopic procedures among low-risk individuals.

This potential role is especially relevant in high-incidence regions where endoscopic resources are limited. In community clinics, primary care settings, or population-level screening programs, standardized tongue-image capture could theoretically be combined with age, sex, symptoms, H. pylori status, family history, blood biomarkers, or questionnaire-based risk factors to generate a risk score. The model would then function as a referral-support tool rather than a diagnostic endpoint. This is consistent with the reviewed evidence, where multimodal models often performed better than image-only models and where the strongest gastric cancer data came from a model that incorporated tongue images with additional biomarkers. However, higher multimodal AUC does not necessarily establish clinically meaningful incremental value from tongue imaging. When clinical or biomarker variables already provide substantial discrimination, future studies should determine whether adding tongue images improves clinically relevant measures such as discrimination, calibration, or decision-making (35).

The current evidence is not sufficient for clinical deployment. Most studies did not report decision thresholds, calibration, decision-curve analysis, net benefit, or downstream clinical consequences. These omissions matter because the acceptable trade-off between missed cancers and unnecessary endoscopies depends on the intended screening context. A model with high AUC may still be clinically unsafe if it is poorly calibrated, if its threshold is chosen *post hoc*, or if it performs poorly in specific subgroups. For this reason, future studies should move beyond reporting discrimination metrics alone and should evaluate whether tongue-image AI improves referral decisions compared with existing risk-stratification approaches.

The evidence base should also be interpreted according to the target condition. Although the review eligibility criteria included related gastrointestinal disorders, the primary clinical question concerns gastric cancer and precancerous gastric lesions. Accordingly, studies addressing GERD, MAFLD, and esophageal cancer were retained as contextual evidence but were not considered direct evidence of gastric cancer diagnostic accuracy. Similarly, the heterogeneous gastrointestinal cohort evaluated by Qiao et al. provides evidence for broader gastrointestinal classification but cannot establish gastric cancer-specific discrimination (34). This distinction is important because disease-specific performance, reference standards, and clinical pathways differ substantially across these conditions.

The contextual studies suggest that tongue-image AI may have broader gastrointestinal applications, but these findings should be regarded as hypothesis-generating rather than as evidence supporting gastric cancer screening.

### 4.4 Methodological and translational challenges

The most important methodological challenge is generalizability. Nearly all included studies were conducted in East Asian populations, predominantly in China, and none of the included models was validated across substantially different ethnic, geographic, dietary, and environmental populations. This concentration of evidence limits confidence in the immediate global applicability of tongue-image AI. Tongue appearance and image characteristics may be influenced by diet, smoking, oral-hygiene practices, medications, systemic diseases, local environmental conditions, microbiome composition, and image-acquisition procedures. These factors may differ between populations and could produce distributional shifts that affect model performance. However, the available literature does not establish that such population differences necessarily reduce diagnostic accuracy; rather, the key limitation is the absence of adequate empirical testing of this assumption. Future studies should therefore prioritize cross-cultural external validation. Models developed in East Asian populations should be prospectively evaluated in independent cohorts from Western, Middle Eastern, South Asian, and other geographically and ethnically diverse populations, preferably using standardized image-acquisition protocols. Such validation should report subgroup-specific discrimination, calibration, sensitivity, specificity, and error patterns rather than relying only on overall AUC. Where performance deterioration is observed, recalibration or retraining should be evaluated transparently. Cross-cultural validation is therefore essential to determine whether models capture disease-related tongue signals or population- and environment-specific visual characteristics (5, 36).

A second challenge is standardization of image acquisition. Dedicated tongue-imaging devices provide controlled lighting and positioning, but their cost and limited availability may restrict implementation. Smartphone-based acquisition is more scalable, but introduces variability in camera sensors, illumination, distance, angle, color reproduction, and patient positioning. Because tongue color and coating are central predictive features, even small differences in acquisition conditions may alter model input and reduce reproducibility. Future studies should therefore report acquisition protocols in detail, include quality-control procedures, evaluate color calibration, and test device-level robustness.

A third challenge is analytic transparency. Several studies used data augmentation, resampling, or cross-validation strategies, but reporting was often insufficient to fully exclude data leakage. For image-based models, augmentation must be applied after patient-level splitting, and images from the same participant should not appear across training and test sets. Similarly, thresholds should be pre-specified or selected within training data only, then evaluated in independent validation data. Without transparent reporting of these procedures, reported performance may be optimistic.

Calibration is another major gap. Most included studies reported AUC, accuracy, sensitivity, and specificity, but few reported calibration curves, Brier scores, calibration slope, calibration-in-the-large, expected calibration error, or recalibration procedures. This is a critical limitation for any model that produces individualized risk estimates. In a screening pathway, an overestimated risk could lead to unnecessary endoscopy, anxiety, and increased healthcare burden, while underestimated risk could delay diagnosis. Discrimination and calibration answer different questions, and both are required before a model can be considered clinically actionable.

### 4.5 Strengths and limitations of this review

This review has several strengths. It used a systematic search strategy across biomedical, multidisciplinary, and engineering databases; applied explicit eligibility criteria; focused on original AI-based tongue-image studies; and synthesized evidence according to target condition, model architecture, validation strategy, performance, interpretability, calibration, and risk of bias. Unlike broader reviews of AI-assisted tongue diagnosis, this review focused specifically on gastric cancer, precancerous gastric lesions, and related gastrointestinal disorders, which allowed a more clinically targeted assessment of the evidence. The review also highlights calibration and external validation as central translational issues, which are often underemphasized in early AI diagnostic studies.

Several limitations should also be acknowledged. First, the number of eligible studies was small, and the target conditions were heterogeneous, preventing formal diagnostic meta-analysis. Second, although multiple databases were searched, unpublished models, regulatory documents, preprints, and non-indexed conference proceedings may have been missed. Third, incomplete reporting in primary studies limited assessment of patient-level splitting, data leakage, threshold selection, calibration, and reproducibility. Finally, because most included studies originated from East Asian populations, the conclusions may not be applicable to other populations or healthcare systems.

### 4.6 Future research directions

Future research should prioritize prospective, multicenter, externally validated studies with predefined analysis plans and clinically meaningful endpoints. For gastric cancer screening, studies should evaluate whether tongue-image AI improves referral decisions compared with standard risk-based approaches and whether it can safely reduce unnecessary endoscopy without increasing missed cancers. For PLGC, larger cohorts with histopathological staging are needed to determine whether tongue-image features can distinguish atrophic gastritis, intestinal metaplasia, dysplasia, and early cancer with sufficient reliability.

The field would also benefit from open benchmark datasets. Publicly available, histopathologically labeled tongue-image datasets with standardized acquisition protocols, expert feature annotations, and metadata on clinical risk factors would enable reproducible model comparison and reduce the current dependence on single-center proprietary datasets. Such datasets should include diverse populations, acquisition devices, and disease stages to support generalizable model development.

Finally, future studies should be designed with clinical translation in mind. This requires reporting not only AUC and accuracy, but also calibration, threshold justification, decision-curve analysis, subgroup performance, fairness assessment, and workflow feasibility. Multimodal models should quantify the incremental value of tongue images beyond conventional risk factors, biomarkers, and symptoms. Only after these steps are completed can tongue-image AI move from proof-of-concept research toward evidence-based clinical implementation.

## 5 Conclusion

Tongue-image artificial intelligence is a promising but early-stage approach for non-invasive screening of gastric cancer, precancerous gastric lesions, and related gastrointestinal disorders. The current evidence suggests that AI models can extract meaningful visual information from tongue images, particularly coating-related features, and that the strongest support comes from multicenter studies of gastric cancer with external validation. However, performance is less robust for precancerous gastric lesions, evidence for other gastrointestinal disorders remains exploratory, and calibration reporting is largely absent. At present, tongue-image AI should be regarded as a potential adjunctive triage tool rather than a replacement for gastroscopy. Prospective external validation, standardized image acquisition, transparent model reporting, calibration assessment, and evaluation in diverse populations are required before clinical deployment can be justified.

## Data Availability

The original contributions presented in the study are included in the article/Supplementary Material, further inquiries can be directed to the corresponding author.
