# Supplementary material for: Artificial intelligence-based tongue image analysis for non-invasive screening of gastric cancer, precancerous lesions, and related gastrointestinal disorders: a systematic review
Source: Front Med (Lausanne). 2026 Sep 29;13:1931256. doi: 10.3389/fmed.2026.1931256 (PMC13658427; doi:10.3389/fmed.2026.1931256)
Supplement: Supplementary file 1 [file Table1.docx]

**Supplementary Material**

**Table S1. PRISMA 2020 checklist**

| **Section and Topic** | **Item #** | **Checklist item** | **Location where item is reported** |
| --- | --- | --- | --- |
| **TITLE** | | |  |
| Title | 1 | Identify the report as a systematic review. | Title |
| **ABSTRACT** | | |  |
| Abstract | 2 | See the PRISMA 2020 for Abstracts checklist. | Abstract |
| **INTRODUCTION** | | |  |
| Rationale | 3 | Describe the rationale for the review in the context of existing knowledge. | Introduction |
| Objectives | 4 | Provide an explicit statement of the objective(s) or question(s) the review addresses. | Introduction; final paragraph before Section 2 |
| **METHODS** | | |  |
| Eligibility criteria | 5 | Specify the inclusion and exclusion criteria for the review and how studies were grouped for the syntheses. | Section 2.2 Eligibility criteria; Section 2.7 Data synthesis |
| Information sources | 6 | Specify all databases, registers, websites, organisations, reference lists and other sources searched or consulted to identify studies. Specify the date when each source was last searched or consulted. | Section 2.3 Information sources and search strategy; Supplementary Tables S1–S2 |
| Search strategy | 7 | Present the full search strategies for all databases, registers and websites, including any filters and limits used. | Section 2.3; Supplementary Tables S1–S2 |
| Selection process | 8 | Specify the methods used to decide whether a study met the inclusion criteria of the review, including how many reviewers screened each record and each report retrieved, whether they worked independently, and if applicable, details of automation tools used in the process. | Section 2.4 Study selection |
| Data collection process | 9 | Specify the methods used to collect data from reports, including how many reviewers collected data from each report, whether they worked independently, any processes for obtaining or confirming data from study investigators, and if applicable, details of automation tools used in the process. | Section 2.5 Data extraction |
| Data items | 10a | List and define all outcomes for which data were sought. Specify whether all results that were compatible with each outcome domain in each study were sought (e.g. for all measures, time points, analyses), and if not, the methods used to decide which results to collect. | Section 2.2 Review question and eligibility criteria; Section 2.5 Data extraction |
|  | 10b | List and define all other variables for which data were sought (e.g. participant and intervention characteristics, funding sources). Describe any assumptions made about any missing or unclear information. | Section 2.5; Sections 2.6 and 2.8 for unclear/missing methodological information |
| Study risk of bias assessment | 11 | Specify the methods used to assess risk of bias in the included studies, including details of the tool(s) used, how many reviewers assessed each study and whether they worked independently, and if applicable, details of automation tools used in the process. | Section 2.6 Risk of bias and applicability assessment; Supplementary Table S4 |
| Effect measures | 12 | Specify for each outcome the effect measure(s) (e.g. risk ratio, mean difference) used in the synthesis or presentation of results. | Sections 2.2 and 2.7; Tables 1–3 |
| Synthesis methods | 13a | Describe the processes used to decide which studies were eligible for each synthesis (e.g. tabulating the study intervention characteristics and comparing against the planned groups for each synthesis (item #5)). | Section 2.7 Data synthesis |
|  | 13b | Describe any methods required to prepare the data for presentation or synthesis, such as handling of missing summary statistics, or data conversions. | Section 2.5 Data extraction |
|  | 13c | Describe any methods used to tabulate or visually display results of individual studies and syntheses. | Sections 2.7 and 3.1–3.6; Tables 1–4; Figures 1–3; Supplementary Tables S4–S5 |
|  | 13d | Describe any methods used to synthesize results and provide a rationale for the choice(s). If meta-analysis was performed, describe the model(s), method(s) to identify the presence and extent of statistical heterogeneity, and software package(s) used. | Section 2.7 Data synthesis |
|  | 13e | Describe any methods used to explore possible causes of heterogeneity among study results (e.g. subgroup analysis, meta-regression). | Section 2.7 Data synthesis; Sections 3.2–3.6 and 4.1–4.5 |
|  | 13f | Describe any sensitivity analyses conducted to assess robustness of the synthesized results. | Not conducted; no sensitivity analysis was reported |
| Reporting bias assessment | 14 | Describe any methods used to assess risk of bias due to missing results in a synthesis (arising from reporting biases). | Not reported; risk of bias due to missing results was not assessed |
| Certainty assessment | 15 | Describe any methods used to assess certainty (or confidence) in the body of evidence for an outcome. | Section 2.8 Strength of evidence assessment; Supplementary Table S5 |
| **RESULTS** | | |  |
| Study selection | 16a | Describe the results of the search and selection process, from the number of records identified in the search to the number of studies included in the review, ideally using a flow diagram. | Figure 1; Section 3.1 Study selection |
|  | 16b | Cite studies that might appear to meet the inclusion criteria, but which were excluded, and explain why they were excluded. | Not reported; excluded reports were summarized by exclusion category rather than cited individually |
| Study characteristics | 17 | Cite each included study and present its characteristics. | Section 3.2 Characteristics of included studies; Table 1 |
| Risk of bias in studies | 18 | Present assessments of risk of bias for each included study. | Section 3.7 Risk of bias, calibration, and reporting quality; Table 4; Supplementary Table S4 |
| Results of individual studies | 19 | For all outcomes, present, for each study: (a) summary statistics for each group (where appropriate) and (b) an effect estimate and its precision (e.g. confidence/credible interval), ideally using structured tables or plots. | Sections 3.3–3.6; Tables 1–3 |
| Results of syntheses | 20a | For each synthesis, briefly summarise the characteristics and risk of bias among contributing studies. | Sections 3.2 and 3.7; Table 4; Supplementary Table S4 |
|  | 20b | Present results of all statistical syntheses conducted. If meta-analysis was done, present for each the summary estimate and its precision (e.g. confidence/credible interval) and measures of statistical heterogeneity. If comparing groups, describe the direction of the effect. | Sections 3.3–3.6; Tables 1–3; no meta-analysis was performed |
|  | 20c | Present results of all investigations of possible causes of heterogeneity among study results. | Section 2.7; Sections 3.2–3.6; Section 4.5 |
|  | 20d | Present results of all sensitivity analyses conducted to assess the robustness of the synthesized results. | Not conducted; no sensitivity analysis was reported |
| Reporting biases | 21 | Present assessments of risk of bias due to missing results (arising from reporting biases) for each synthesis assessed. | Not reported; risk of bias due to missing results was not assessed |
| Certainty of evidence | 22 | Present assessments of certainty (or confidence) in the body of evidence for each outcome assessed. | Section 2.8 Strength of evidence assessment; Section 3.7; Supplementary Table S5 |
| **DISCUSSION** | | |  |
| Discussion | 23a | Provide a general interpretation of the results in the context of other evidence. | Discussion, Sections 4.1–4.6 |
|  | 23b | Discuss any limitations of the evidence included in the review. | Discussion, Section 4.5 Strengths and limitations of this review |
|  | 23c | Discuss any limitations of the review processes used. | Discussion, Section 4.5 Strengths and limitations of this review |
|  | 23d | Discuss implications of the results for practice, policy, and future research. | Discussion, Section 4.6 Future research directions; Section 5 Conclusion |
| **OTHER INFORMATION** | | |  |
| Registration and protocol | 24a | Provide registration information for the review, including register name and registration number, or state that the review was not registered. | Section 2.1 Reporting framework and protocol; PROSPERO registration CRD420261444857 |
|  | 24b | Indicate where the review protocol can be accessed, or state that a protocol was not prepared. | Section 2.1; full protocol not publicly available |
|  | 24c | Describe and explain any amendments to information provided at registration or in the protocol. | Section 2.1 Reporting framework and protocol |
| Support | 25 | Describe sources of financial or non-financial support for the review, and the role of the funders or sponsors in the review. | Funding statement |
| Competing interests | 26 | Declare any competing interests of review authors. | Conflict of Interest statement |
| Availability of data, code and other materials | 27 | Report which of the following are publicly available and where they can be found: template data collection forms; data extracted from included studies; data used for all analyses; analytic code; any other materials used in the review. | Data availability statement; Supplementary Tables S1–S5 |

*From:*  Page MJ, McKenzie JE, Bossuyt PM, Boutron I, Hoffmann TC, Mulrow CD, et al. The PRISMA 2020 statement: an updated guideline for reporting systematic reviews. BMJ 2021;372:n71. doi: 10.1136/bmj.n71. This work is licensed under CC BY 4.0. To view a copy of this license, visit <https://creativecommons.org/licenses/by/4.0/>

**Table S2. Search terms used for systematic search**

| **PIRD component** | **Population / target condition** | **Index test** | **Model / method** |
| --- | --- | --- | --- |
| **Concept** | Gastric cancer, precancerous gastric lesions, and related gastrointestinal disorders | Tongue image analysis and tongue diagnosis | Artificial intelligence, machine learning, deep learning, and image-analysis methods |
| **Search terms** | gastric cancer; stomach cancer; gastric neoplasm; stomach neoplasm; gastric tumor; stomach tumor; precancerous gastric lesion; PLGC; gastric precancerous lesion; atrophic gastritis; intestinal metaplasia; dysplasia; gastritis; gastrointestinal disease; digestive disease; upper gastrointestinal disease; gastroesophageal reflux; GERD; metabolic-associated fatty liver disease; MAFLD; esophageal cancer; oesophageal cancer; esophageal neoplasm; premalignant lesion | tongue image; tongue images; tongue imaging; tongue diagnosis; tongue inspection; computerized tongue inspection; computer-aided tongue diagnosis; digital tongue; tongue coating; tongue coat; tongue fur; tongue body; tongue color; tongue colour; tongue feature; tongue features; tongue texture; tongue morphology; tongue photograph; tongue photo; TCM tongue | artificial intelligence; AI; machine learning; deep learning; neural network; convolutional neural network; CNN; ResNet; EfficientNet; transformer; vision transformer; Swin Transformer; computer vision; image analysis; image classification; feature extraction; segmentation; computer-aided diagnosis; computer assisted diagnosis; diagnostic model; prediction model; classification model; XGBoost; random forest; support vector machine; SVM |

**Table S3. Core Boolean blocks for each database**

| **Database** | **Block** | **Exact search query** | **Results** |
| --- | --- | --- | --- |
| PubMed | #1 | "Tongue"[Mesh] OR "tongue image*"[tiab] OR "tongue imaging"[tiab] OR "tongue diagnosis"[tiab] OR "tongue inspection"[tiab] OR "computerized tongue"[tiab] OR "computer-aided tongue diagnosis"[tiab] OR "digital tongue"[tiab] OR "tongue coating"[tiab] OR "tongue coat"[tiab] OR "tongue fur"[tiab] OR "tongue body"[tiab] OR "tongue color"[tiab] OR "tongue colour"[tiab] OR "tongue feature*"[tiab] OR "tongue texture"[tiab] OR "tongue morphology"[tiab] OR "tongue photograph*"[tiab] OR "tongue photo*"[tiab] OR "TCM tongue"[tiab] | 25,777 |
|  | #2 | "Artificial Intelligence"[Mesh] OR "Machine Learning"[Mesh] OR "Deep Learning"[Mesh] OR "Diagnosis, Computer-Assisted"[Mesh] OR "artificial intelligence"[tiab] OR AI[tiab] OR "machine learning"[tiab] OR "deep learning"[tiab] OR "neural network*"[tiab] OR CNN[tiab] OR "convolutional neural network*"[tiab] OR ResNet[tiab] OR EfficientNet[tiab] OR transformer*[tiab] OR "vision transformer*"[tiab] OR "Swin Transformer"[tiab] OR "computer vision"[tiab] OR "image analysis"[tiab] OR "image classification"[tiab] OR "feature extraction"[tiab] OR segmentation[tiab] OR "computer-aided diagnosis"[tiab] OR "computer assisted diagnosis"[tiab] OR "diagnostic model*"[tiab] OR "prediction model*"[tiab] OR "classification model*"[tiab] OR XGBoost[tiab] OR "random forest"[tiab] OR SVM[tiab] OR "support vector machine"[tiab] | 854,743 |
|  | #3 | "Stomach Neoplasms"[Mesh] OR "Gastrointestinal Diseases"[Mesh] OR "Gastritis"[Mesh] OR "Gastroesophageal Reflux"[Mesh] OR "Esophageal Neoplasms"[Mesh] OR "Fatty Liver"[Mesh] OR "gastric cancer"[tiab] OR "stomach cancer"[tiab] OR "gastric neoplasm*"[tiab] OR "stomach neoplasm*"[tiab] OR "gastric tumor*"[tiab] OR "stomach tumor*"[tiab] OR "precancerous gastric lesion*"[tiab] OR "gastric precancerous lesion*"[tiab] OR PLGC[tiab] OR "atrophic gastritis"[tiab] OR "intestinal metaplasia"[tiab] OR dysplasia[tiab] OR gastritis[tiab] OR "gastrointestinal disease*"[tiab] OR "digestive disease*"[tiab] OR "upper gastrointestinal"[tiab] OR GERD[tiab] OR "gastroesophageal reflux"[tiab] OR "metabolic-associated fatty liver disease"[tiab] OR MAFLD[tiab] OR "esophageal cancer"[tiab] OR "oesophageal cancer"[tiab] OR "esophageal neoplasm*"[tiab] OR "premalignant lesion*"[tiab] | 1,385,251 |
|  | #4 | #1 AND #2 AND #3 | 41 |
| Web of Science | #5 | TS=("tongue image*" OR "tongue imaging" OR "tongue diagnosis" OR "tongue inspection" OR "computerized tongue" OR "computer-aided tongue diagnosis" OR "digital tongue" OR "tongue coating" OR "tongue coat" OR "tongue fur" OR "tongue body" OR "tongue color" OR "tongue colour" OR "tongue feature*" OR "tongue texture" OR "tongue morphology" OR "tongue photograph*" OR "tongue photo*" OR "TCM tongue") | [1,968](https://www.webofscience.com/wos/woscc/summary/e6f763e8-74b5-42c1-986b-1aea2c1d5882-01bd7ed3e3/relevance/1) |
|  | #6 | TS=("artificial intelligence" OR AI OR "machine learning" OR "deep learning" OR "neural network*" OR CNN OR "convolutional neural network*" OR ResNet OR EfficientNet OR transformer* OR "vision transformer*" OR "Swin Transformer" OR "computer vision" OR "image analysis" OR "image classification" OR "feature extraction" OR segmentation OR "computer-aided diagnosis" OR "computer assisted diagnosis" OR "diagnostic model*" OR "prediction model*" OR "classification model*" OR XGBoost OR "random forest" OR SVM OR "support vector machine") | [2,917,464](https://www.webofscience.com/wos/woscc/summary/f97d8688-ac73-4ed0-905a-5ad51f7bfd33-01bd7ed7d8/relevance/1) |
|  | #7 | TS=("gastric cancer" OR "stomach cancer" OR "gastric neoplasm*" OR "stomach neoplasm*" OR "gastric tumor*" OR "stomach tumor*" OR "precancerous gastric lesion*" OR "gastric precancerous lesion*" OR PLGC OR "atrophic gastritis" OR "intestinal metaplasia" OR dysplasia OR gastritis OR "gastrointestinal disease*" OR "digestive disease*" OR "upper gastrointestinal" OR GERD OR "gastroesophageal reflux" OR "metabolic-associated fatty liver disease" OR MAFLD OR "esophageal cancer" OR "oesophageal cancer" OR "esophageal neoplasm*" OR "premalignant lesion*") | [399,402](https://www.webofscience.com/wos/woscc/summary/65bcffd8-6061-4415-98c0-86eba011c2ad-01bd7edad4/relevance/1) |
|  | #8 | #5 AND #6 AND #7 | [39](https://www.webofscience.com/wos/woscc/summary/8bef9c40-e2d8-43af-9bb7-061f1a7dba4d-01bd7ee193/relevance/1) |
| Scopus | #9 | TITLE-ABS-KEY("tongue image*" OR "tongue imaging" OR "tongue diagnosis" OR "tongue inspection" OR "computerized tongue" OR "computer-aided tongue diagnosis" OR "digital tongue" OR "tongue coating" OR "tongue coat" OR "tongue fur" OR "tongue body" OR "tongue color" OR "tongue colour" OR "tongue feature*" OR "tongue texture" OR "tongue morphology" OR "tongue photograph*" OR "tongue photo*" OR "TCM tongue") | 2,854 |
|  | #10 | TITLE-ABS-KEY("artificial intelligence" OR AI OR "machine learning" OR "deep learning" OR "neural network*" OR CNN OR "convolutional neural network*" OR ResNet OR EfficientNet OR transformer* OR "vision transformer*" OR "Swin Transformer" OR "computer vision" OR "image analysis" OR "image classification" OR "feature extraction" OR segmentation OR "computer-aided diagnosis" OR "computer assisted diagnosis" OR "diagnostic model*" OR "prediction model*" OR "classification model*" OR XGBoost OR "random forest" OR SVM OR "support vector machine") | 4,931,440 |
|  | #11 | TITLE-ABS-KEY("gastric cancer" OR "stomach cancer" OR "gastric neoplasm*" OR "stomach neoplasm*" OR "gastric tumor*" OR "stomach tumor*" OR "precancerous gastric lesion*" OR "gastric precancerous lesion*" OR PLGC OR "atrophic gastritis" OR "intestinal metaplasia" OR dysplasia OR gastritis OR "gastrointestinal disease*" OR "digestive disease*" OR "upper gastrointestinal" OR GERD OR "gastroesophageal reflux" OR "metabolic-associated fatty liver disease" OR MAFLD OR "esophageal cancer" OR "oesophageal cancer" OR "esophageal neoplasm*" OR "premalignant lesion*") | 682,366 |
|  | #12 | #9 AND #10 AND #11 | 59 |
| IEEE Xplore | #13 | ("All Metadata":("tongue image*" OR "tongue imaging" OR "tongue diagnosis" OR "tongue inspection" OR "computerized tongue" OR "computer-aided tongue diagnosis" OR "digital tongue" OR "tongue coating" OR "tongue coat" OR "tongue fur" OR "tongue body" OR "tongue color" OR "tongue colour" OR "tongue feature*" OR "tongue texture" OR "tongue morphology" OR "tongue photograph*" OR "tongue photo*" OR "TCM tongue")) AND ("All Metadata":("artificial intelligence" OR AI OR "machine learning" OR "deep learning" OR "neural network*" OR CNN OR "convolutional neural network*" OR ResNet OR EfficientNet OR transformer* OR "vision transformer*" OR "Swin Transformer" OR "computer vision" OR "image analysis" OR "image classification" OR "feature extraction" OR segmentation OR "computer-aided diagnosis" OR "computer assisted diagnosis" OR "diagnostic model*" OR "prediction model*" OR "classification model*" OR XGBoost OR "random forest" OR SVM OR "support vector machine")) AND ("All Metadata":("gastric cancer" OR "stomach cancer" OR "gastric neoplasm*" OR "stomach neoplasm*" OR "gastric tumor*" OR "stomach tumor*" OR "precancerous gastric lesion*" OR "gastric precancerous lesion*" OR PLGC OR "atrophic gastritis" OR "intestinal metaplasia" OR dysplasia OR gastritis OR "gastrointestinal disease*" OR "digestive disease*" OR "upper gastrointestinal" OR GERD OR "gastroesophageal reflux" OR "metabolic-associated fatty liver disease" OR MAFLD OR "esophageal cancer" OR "oesophageal cancer" OR "esophageal neoplasm*" OR "premalignant lesion*")) | 0 |

**Table S4. Structured certainty assessment of the evidence**

| **Evidence group / study** | **Risk of bias** | **Indirectness** | **Imprecision** | **External validity** | **Overall contribution / rationale** |
| --- | --- | --- | --- | --- | --- |
| **Gastric cancer** | | | | | |
| Zhu et al. (2022) | High | Moderate | High | High concern | Small case-control study; participant-level splitting and augmentation sequence unclear; no external validation; no 95% CI reported; highly selected population and specialized image-acquisition conditions. |
| Yuan et al. (2023) | Low | Low | Low | Low concern | Prospective multicenter study with histopathological reference standards and geographically independent external validation; 95% CIs reported for external AUCs. Main limitation was limited formal calibration assessment. |
| Qiao et al. (2024) | Moderate | Moderate | High | Moderate concern | Participant-level splitting and leakage prevention were appropriately reported, but the study included heterogeneous gastrointestinal disease categories, used mixed reference standards, lacked external validation and did not report 95% CIs. Its contribution to GC-specific conclusions is therefore indirect/limited. |
| **Precancerous gastric lesions / high-risk gastric screening** | | | | | |
| Ma et al. (2023) | Moderate | Low | High | Moderate concern | Histopathological confirmation and external validation strengthen the evidence, but the development cohort had a relatively small PLGC sample, and performance CIs and calibration were not adequately reported. |
| Wang et al. (2024) | Low–moderate | Low | Low | Moderate concern | External validation was performed, but performance decreased in the external cohort; no calibration assessment or 95% CIs for the principal performance estimates were reported. |
| Yu et al. (2025) | Moderate | Low | Moderate | Moderate concern | Multicenter cohort with appropriate gastric reference standards and a test set, but no dedicated external validation for the image-based model and limited precision reporting for some model estimates. |
| **Contextual evidence** | | | | | |
| Wu et al. (2020) | Moderate | High | Low | High concern | Small single-center GERD case-control study with modest discrimination. The target condition is outside the primary GC/PLGC question, although 95% CIs were reported. |
| Dai et al. (2024) | High | High | High | High concern | Small single-center MAFLD study with potential confounding, possible leakage concerns, no performance CIs, and a target condition outside the primary gastric evidence base. |
| Jin/Duan et al. (2018) | Low–moderate | High | High | High concern | Large screening cohort with histological confirmation, but the target condition was esophageal cancer/premalignant lesions, there was no external validation, and key performance CIs were not reported. |
| **Overall certainty** | | | | | |
| gastric cancer evidence (Zhu, Yuan, Qiao) | Moderate concerns | Moderate | Moderate | Moderate | Low certainty |
| PLGC/high-risk gastric screening (Ma, Wang, Yu) | Moderate concerns | Low | Moderate | Moderate | Low certainty |
| contextual evidence (Wu, Dai, Jin/Duan) | Moderate concerns | High | High | High | Very low certainty |

**Table S5. Signaling questions and study-level judgments for risk of bias and applicability**

| **Study** | **Domain / signaling question** | **Judgment** | **Study-level reason** |
| --- | --- | --- | --- |
| Zhu et al., 2023 | Analysis: Was data splitting performed at the participant level? | Unclear | The study reported an 8:1:1 split of images, but did not establish that all images from the same participant were restricted to one partition. |
|  | Analysis: Was augmentation performed after dataset splitting and restricted to the development/training data? | Unclear | Geometric transformations and Gaussian-noise augmentation were used, but the timing of augmentation relative to dataset splitting was not reported. |
|  | Analysis: Was model development separated from final performance assessment? | Low risk | Separate training, validation, and test sets were reported; however, the dataset used for preliminary feature selection was not clearly specified. |
|  | Analysis: Was class imbalance appropriately handled without introducing leakage? | Unclear | Augmentation was used to expand the dataset because of class imbalance, but whether this was restricted to the training set was not reported. |
|  | Analysis: Was an independent external validation performed? | High concern | No external validation was reported. |
|  | Analysis: Overall judgment | High risk | Participant-level separation and augmentation timing were not established, creating potential leakage risk; external validation was also absent. |
|  | Applicability: Does the study population represent the intended clinical-use population? | High concern | The study used a highly selected case-control sample consisting of gastric-cancer and non-gastric-cancer subjects rather than a representative pre-endoscopic screening population. |
|  | Applicability: Is the target condition appropriate to the review question? | Low concern | The target condition was gastric cancer, directly matching the primary review question. |
|  | Applicability: Is the index test representative of intended clinical use? | High concern | Tongue images were acquired using a dedicated/bespoke acquisition system under controlled conditions, limiting direct generalizability to routine clinical screening. |
|  | Applicability: Overall judgment | High concern | The target condition is directly relevant, but the highly selected population and specialized image-acquisition conditions limit applicability to real-world screening. |
| Yuan et al., 2023 | Analysis: Was data splitting performed at the participant level? | Low risk | Cases were allocated to the development cohort and a separate geographically distinct external validation cohort. |
|  | Analysis: Was model development separated from final performance assessment? | Low risk | Approximately 80% of cases were used for training and 20% for internal validation, followed by testing in an independent external cohort. |
|  | Analysis: Was an independent external validation performed? | Low risk | An independent geographically distinct external validation cohort was used. |
|  | Analysis: Was calibration appropriately assessed? | Unclear | Probability-distribution plots were reported, but a formal calibration measure such as calibration slope, intercept, Brier score, or ECE was not reported. |
|  | Analysis: Overall judgment | Low concern | Large prospective multicentre design, participant-level cohort allocation, and independent geographic validation support low analysis concern. |
|  | Applicability: Does the study population represent the intended clinical-use population? | Low concern | The prospective multicentre cohort included patients undergoing clinical evaluation and a range of non-gastric-cancer controls. |
|  | Applicability: Is the target condition appropriate to the review question? | Low concern | Gastric cancer was the primary target condition. |
|  | Applicability: Is the index test representative of intended clinical use? | Low concern | Tongue imaging was evaluated in a clinical setting and incorporated into a screening/diagnostic framework. |
|  | Applicability: Overall judgment | Low concern | The population, target condition, and clinical application closely correspond to the intended use of the technology. |
| Qiao et al., 2024 | Analysis: Was data splitting performed at the participant level? | Low risk | Participant-level separation was explicitly described. |
|  | Analysis: Was augmentation/resampling restricted to development data? | Low risk | Resampling and augmentation were reported within the training/development procedure. |
|  | Analysis: Was an independent external validation performed? | High concern | No independent external validation was reported. |
|  | Analysis: Overall judgment | Low concern | Participant-level separation and training-data resampling reduced leakage concerns, although external validation was absent. |
|  | Applicability: Does the study population represent the intended clinical-use population? | High concern | The study included patients with several gastrointestinal conditions and controls rather than a population specifically representative of gastric-cancer screening. |
|  | Applicability: Is the target condition appropriate to the review question? | High concern | The primary targets were related gastrointestinal disorders rather than gastric cancer or precancerous gastric lesions. |
|  | Applicability: Is the index test representative of intended clinical use? | Low concern | Tongue images were acquired using a smartphone-based approach that is relatively compatible with routine clinical use. |
|  | Applicability: Overall judgment | High concern | The image-acquisition approach is clinically feasible, but the target conditions are outside the primary gastric-cancer/PLGC question. |
| Ma et al., 2023 | Analysis: Was data splitting performed at the participant level? | Low risk | Development and validation cohorts were separated geographically/hospital-wise. |
|  | Analysis: Was an independent external validation performed? | Low risk | A large independent validation cohort was used. |
|  | Analysis: Was sample size adequately justified? | Unclear | No formal sample-size calculation was reported for the relatively small development cohort. |
|  | Analysis: Was calibration assessed? | High concern | No formal calibration assessment was reported. |
|  | Analysis: Overall judgment | Low concern | Participant-level cohort separation and independent external validation were strengths, although sample-size justification and calibration reporting were limited. |
|  | Applicability: Does the study population represent the intended clinical-use population? | Low concern | The population consisted of patients undergoing clinical evaluation for gastric lesions. |
|  | Applicability: Is the target condition appropriate to the review question? | Low concern | The study directly addressed precancerous gastric lesions. |
|  | Applicability: Is the reference standard appropriate? | Low concern | Histopathological assessment provided an appropriate reference standard. |
|  | Applicability: Overall judgment | Low concern | The target condition and reference standard are directly relevant to pre-endoscopic PLGC risk stratification. |
| Wang et al., 2024 | Analysis: Was data splitting performed at the participant level? | Low risk | The authors explicitly stated that all images from each patient were allocated to the same cohort. |
|  | Analysis: Was augmentation restricted to the training procedure? | Low risk | Random cropping, flipping, and rotation were applied during neural-network training after cohort allocation. |
|  | Analysis: Was an independent external validation performed? | Low risk | A separate geographically distinct external validation cohort was reported. |
|  | Analysis: Was calibration assessed? | High concern | No formal calibration assessment was reported. |
|  | Analysis: Overall judgment | Low concern | Explicit participant-level separation, training-stage augmentation, and external validation support low analysis concern. |
|  | Applicability: Does the study population represent the intended clinical-use population? | Low concern | The study involved clinically evaluated patients with gastric lesions. |
|  | Applicability: Is the target condition appropriate? | Low concern | The target was PLGC risk classification. |
|  | Applicability: Is the index test representative of intended clinical use? | Low concern | The tongue-imaging approach was evaluated in clinical cohorts and externally validated. |
|  | Applicability: Overall judgment | Low concern | Population, target condition, and validation setting were clinically relevant. |
| Yu et al., 2025 | Analysis: Was data splitting performed at the participant level? | Unclear | A 70:30 split was reported, but the report did not explicitly establish that multiple images from one participant could not occur in both partitions. |
|  | Analysis: Was augmentation performed after splitting? | Unclear | Augmentation was described, but its timing relative to the 70:30 split was not clearly reported. |
|  | Analysis: Was an independent external validation performed? | Concern | No dedicated independent external validation of the image model was reported. |
|  | Analysis: Overall judgment | Low concern | The available information did not demonstrate leakage or major analytical bias, although participant-level separation and augmentation timing were incompletely reported. |
|  | Applicability: Does the study population represent the intended clinical-use population? | Low concern | The study addressed gastric cancer, although its case-control elements and selected healthy participants limit representativeness. |
|  | Applicability: Is the target condition appropriate? | Low concern | Gastric cancer was directly evaluated. |
|  | Applicability: Is the index test representative of intended clinical use? | Low concern | Tongue imaging was used as the principal non-invasive input. |
|  | Applicability: Overall judgment | Low concern | Despite selection limitations, the target condition and image-based application are directly relevant to the review question. |
| Wu et al., 2020 | Analysis: Was participant-level separation relevant and appropriately handled? | Low concern | The study was a clinical observational analysis rather than a train/test deep-learning study; participant-level data were analyzed as individual observations. |
|  | Analysis: Was sample size justified? | Low concern | A formal sample-size calculation was reported. |
|  | Analysis: Was external validation performed? | High concern | No external validation was reported. |
|  | Analysis: Was calibration assessed? | High concern | No formal calibration assessment was reported. |
|  | Analysis: Overall judgment | Low concern | The study used a predefined clinical feature set and a justified sample size, although it lacked external validation and calibration assessment. |
|  | Applicability: Does the study population represent the intended clinical-use population? | High concern | The study used a selected GERD case-control population with asymptomatic controls and stringent exclusions. |
|  | Applicability: Is the target condition appropriate? | High concern | GERD is a related gastrointestinal condition rather than gastric cancer or PLGC. |
|  | Applicability: Is the index test representative of intended clinical use? | High concern | A specialized automatic tongue-diagnosis system was used under controlled image-acquisition conditions. |
|  | Applicability: Overall judgment | High concern | The target condition and selected population substantially limit direct applicability to gastric-cancer screening. |
| Dai et al., 2024 | Analysis: Was data splitting performed at the participant level? | High risk | The Tongue-GAN component used 1,600 training and 400 test images, but participant-level separation was not established. |
|  | Analysis: Could images from the same participant enter different partitions? | High risk | Multiple images originated from a large standardized tongue-image database, while participant-level allocation was not reported. |
|  | Analysis: Was augmentation restricted to development data? | Unclear | Clipping, flipping, and normalization were reported, but their timing relative to dataset splitting was not specified. |
|  | Analysis: Was the test set used for model selection? | High risk | The report states that model weights with the highest test-set accuracy were selected, indicating use of the test set during model development. |
|  | Analysis: Was external validation performed? | High concern | No independent external validation was reported. |
|  | Analysis: Overall judgment | High concern | Image-level splitting and explicit use of test-set performance for model-weight selection create substantial risk of optimistic bias. |
|  | Applicability: Does the study population represent the intended clinical-use population? | High concern | MAFLD cases were compared with selected healthy volunteers, including students, with substantial age and selection differences. |
|  | Applicability: Is the target condition appropriate? | High concern | The target was MAFLD, a liver condition outside the primary gastric-cancer/PLGC question. |
|  | Applicability: Does the model require information available at screening? | High concern | The multimodal model required microbiota sequencing and additional clinical variables, which are not representative of simple tongue-image screening. |
|  | Applicability: Overall judgment | High concern | The target condition, selected population, and impractical multimodal inputs substantially limit applicability to the intended clinical use. |
| Duan/Jin et al., 2018 | Analysis: Was data splitting performed at the participant level? | Low concern | Cases were divided into training and test groups at the participant level. |
|  | Analysis: Was augmentation/resampling used? | Low concern | No image augmentation or class-imbalance resampling was reported. |
|  | Analysis: Was an independent external validation performed? | High concern | No independent external validation was reported. |
|  | Analysis: Overall judgment | Low concern | Participant-level grouping and prospective screening-based testing support low analysis concern, although external validation was absent. |
|  | Applicability: Does the study population represent the intended clinical-use population? | Low concern | The study included participants from a primary screening setting. |
|  | Applicability: Is the target condition appropriate? | High concern | The target was esophageal cancer and premalignant lesions rather than gastric cancer or PLGC. |
|  | Applicability: Is the reference standard appropriate? | Low concern | Histological confirmation was used for the disease outcomes. |
|  | Applicability: Overall judgment | High concern | The screening-based design and strong reference standard support clinical applicability, but the target condition differs from the primary gastric-cancer question. |
